# Optimizing therapeutic outcomes with Mechanotherapy and Ultrasound Sonopermeation in solid tumors

**DOI:** 10.1371/journal.pcbi.1012676

**Published:** 2025-09-23

**Authors:** Marina Koutsi, Triantafyllos Stylianopoulos, Fotios Mpekris

**Affiliations:** 1 Cancer Biophysics Laboratory, Department of Mechanical and Manufacturing Engineering, University of Cyprus, Nicosia, Cyprus; 2 Cancer Genetics, Therapeutics & Ultrastructural Pathology Department, The Cyprus Institute of Neurology and Genetics, Nicosia, Cyprus; Universidad de Granada, SPAIN

## Abstract

Mechanical solid stress plays a pivotal role in tumor progression and therapeutic response. Elevated solid stress compresses intratumoral blood vessels, leading to hypoperfusion, and hypoxia, which impair oxygen and drug delivery. These conditions hinder the efficacy of drugs and promote tumor progression and treatment resistance compromising therapeutic outcomes. To enhance treatment efficacy, mechanotherapeutics and ultrasound sonopermeation have been developed to improve tumor perfusion and drug delivery. Mechanotherapy aims to reduce tumor stiffness and mechanical stress within tumors to normal levels leading to decompression of vessels while simultaneously improving perfusion. On the other hand, ultrasound sonopermeation strategy focuses on increasing non-invasively and transiently tumor vessel wall permeability to boost perfusion and thus, improve drug delivery. Within this framework and aiming to replicate published experimental data in silico, we developed a mathematical model designed to derive optimal conditions for the combined use of mechanotherapeutics and sonopermeation, with the goal of optimizing efficacy of nano-immunotherapy. The model incorporates complex interactions among diverse components that are crucial in the multifaceted process of tumor progression. These components encompass a variety of cell populations in tumor, such as tumor cells and immune cells, as well as components of the tumor vasculature including endothelial cells, angiopoietins, and the vascular endothelial growth factor. Seeking initial model verification, we carried out validation of model predictions with published experimental data, wherein a strong correlation was observed between the model predictions and the actual experimental measurements of critical parameters, which are essential to reinforce the overall accuracy of the mathematical framework employed. In addition, a parametric analysis was performed with primary objective to investigate the impact of various critical parameters that influence sonopermeation. Model predictions showed maximal drug delivery and tumor volume reduction at an acoustic pressure range of 0.24–0.27 MPa and mechanical index of 0.17, consistent with values used in clinical trials following sonopermeation treatment. The analysis provided optimal guidelines for the use of sonopermeation in conjunction with mechanotherapy, that contribute to identify optimal conditions for sonopermeation.

## Author summary

Solid tumors consist not only of malignant cells but also of stromal cells and an extracellular matrix, collectively shaping a complex tumor microenvironment. In tumors with significant fibrosis, excessive extracellular matrix accumulation—primarily collagen and hyaluronan—results in increased rigidity and mechanical solid stress accumulation. This stress can compress blood vessels, leading to reduced blood flow (hypoperfusion) and oxygen deprivation (hypoxia), which in turn impede drug delivery and fuel tumor progression. Mechanotherapeutic approaches focus on alleviating tumor stiffness and solid stress by targeting extracellular matrix components or Cancer-Associated Fibroblasts, ultimately relieving vessel compression and enhancing perfusion. Ultrasound sonopermeation, a technique combining ultrasound waves with microbubbles, has been explored to temporarily increase vascular permeability and alleviate intratumoral solid stress, thereby facilitating drug penetration. Recent experimental studies indicate that integrating mechanotherapy with sonopermeation may yield synergistic benefits, further enhancing treatment effectiveness. Here, we developed a mathematical model to optimize these combined therapeutic strategies by incorporating crucial tumor microenvironmental dynamics. The model provides results for the most effective treatment conditions, aligning closely with experimental and clinical findings. Additionally, we performed a parametric analysis to determine the optimal values of ultrasound frequency and mechanical index required for sonopermeation to maximize drug delivery and improve the overall efficacy of cancer therapy.

## 1. Introduction

Solid tumors are complex biological entities that are composed not solely of malignant cellular populations but also include various additional components, such as stromal cells and extracellular matrix (ECM), which collectively form the tumor microenvironment (TME) [1–5]. In numerous instances of tumor pathophysiology, particularly in highly desmoplastic cancers such as various sarcoma subtypes, the TME becomes fibrotic as the tumor proliferates. This fibrosis is indicative of an augmented synthesis of ECM elements, predominantly collagen and hyaluronan, which contribute to the development of a tumor mass with elevated stiffness, thereby impacting its mechanical properties [6]. The high density of cancer cells, stromal components, and extracellular matrix elements, coupled with the accelerated growth of the tumor at the expense of surrounding host tissue, generates mechanical forces referred to as solid stress, which manifest both within the tumor and between the tumor and surrounding host tissue [7,8]. The role of mechanical solid stresses is critical in influencing the progression of tumors as well as the efficacy of therapeutic interventions [9,10]. The elevation of solid stress can induce compression of intratumoral blood vessels and ultimately result in their collapse and dysfunction, thereby compromising the delivery of oxygen and nutrients to the tumor mass, leading to hypoperfusion and hypoxia [11–13]. Hypoperfusion can severely hinder the effective intratumoral delivery of therapeutics administered systemically, while the prevailing hypoxic environment can significantly enhance tumor progression and confer resistance to treatment through a variety of mechanisms [14–16].

A therapeutic strategy aimed at decompressing vessels while simultaneously enhancing the perfusion levels within tumor tissues involves the application of mechanotherapeutics, which are intended to reduce tumor stiffness and the mechanical forces within tumors to normal levels [17]. This is achieved by specifically targeting ECM components, such as collagen and hyaluronan, or Cancer-Associated Fibroblasts (CAFs), thereby reopening compressed blood vessels and improving both the perfusion and distribution of therapeutic drugs within the TME [18–26]. It was demonstrated both mathematically and experimentally that the mechanotherapeutic agent tranilast, typically utilized as an anti-fibrotic drug, employs a stress alleviation strategy that enhances the functional vascular density by decompressing blood vessels and improving the delivery of therapeutic agents [20,27]. Ketotifen, an antihistamine medication, has been effectively demonstrated that possesses a dual functionality, whereby it serves as both a mechanomodulator and an immunomodulator within the TME specifically in sarcomas [6,28,29]. Nevertheless, it is important to note that the efficacy of mechanotherapeutics is somewhat limited, as they are only able to decompress a fraction of the compressed blood vessels within the tumor, rather than achieving comprehensive decompression of all vessels [23]. This limited therapeutic efficacy primarily stems from the heterogeneous characteristics of the tumor microenvironment. For example, losartan mitigates solid stress by acting on cancer-associated fibroblasts (CAFs) and downregulating signaling molecules such as TGF-β1 and CCN2, which drive the production of collagen and hyaluronan. These extracellular matrix components work synergistically to generate solid stress that compresses tumor blood vessels [12,23,30]. Losartan tends to be most effective in tumors with high levels of both collagen and hyaluronan. Conversely, in tumors with low desmoplasia, the impact of losartan and similar mechanotherapeutics is significantly reduced [16,23,26,31,32]. Additionally, most mechanotherapeutics are repurposed drugs that, while already approved for clinical use, carry their own side effects. For instance, losartan is a potent antihypertensive agent, which limits the ability to increase its dosage for achieving greater vascular decompression.

Ultrasound sonopermeation is a method to enhance drug delivery in solid tumors that utilizes ultrasound in combination with microbubbles. Sonopermeation can enhance transiently the permeability of vessel walls, thereby improving the delivery of therapeutic agents. This method has shown promise in overcoming biological barriers and improving drug delivery to tumors [33,34]. While this approach is effective for improving drug uptake, its direct impact on tumor cells remains limited. However, several experimental studies have demonstrated that ultrasound sonopermeation cannot induce apoptosis in cancer cells, especially at low acoustic pressures [35–37]. It has been also found that sonopermeation can reduce intratumoral solid stress and thus, improve perfusion. However, the underlying mechanisms through which ultrasound and microbubble interactions lead to a reduction in solid stress remain inadequately understood, particularly in terms of how physical forces translate into changes in the tumor stroma and extracellular matrix structure [38,39]. Indeed, the use of ultrasound in the presence of microbubbles has exhibited enhanced therapeutic efficacy when compared to traditional nano- and chemo-therapeutic agents [34,40–44]. Interestingly, we provided evidence that mechanotherapy can be combined with sonopermeation and that the two strategies can have synergistic effects on improving therapeutic efficacy [6].

Up to now, there has been limited research on mathematical modeling related to sonopermeation and drug delivery for the treatment of solid tumors. Few mathematical models have been formulated to investigate the impact of microbubbles within blood vessels [45,46], often overlooking perfusion and drug delivery challenges posed by the TME and lacking experimental validation. Recently, a more detailed mathematical model was developed to describe the mechanisms by which low-intensity ultrasound can serve to inhibit the proliferation and expansion of stem-like cancer cells [47]. Specifically, Blanco et al. [47–49], investigated complex interactions of mechanical forces, cellular reactions, and the dynamic progression of tumors over time and space. In the present study, we developed a model for the study of the combined effects of mechanotherapy and sonopermeation, building on our previous studies [27,50–53]. More precisely, the newly developed model has been designed to derive optimal conditions for the combined use of mechanotherapeutics and sonopermeation, by integrating the crucial effects of sonopermeation, alongside the impact of the mechanotherapeutic ketotifen on the tumor microenvironment. Furthermore, a comparison of model predictions with experimental data was carried out to substantiate the predictions generated by the mathematical model. Finally, a parametric analysis was performed with the primary objective of investigating the impact of various critical parameters that significantly influence the effects of sonopermeation, which in turn aims to enhance our understanding on the mechanism by which sonopermeation can enhance cancer treatment.

## 2. Materials and methods I

### 2.1. Description of the mathematical model

**Fig 1:**
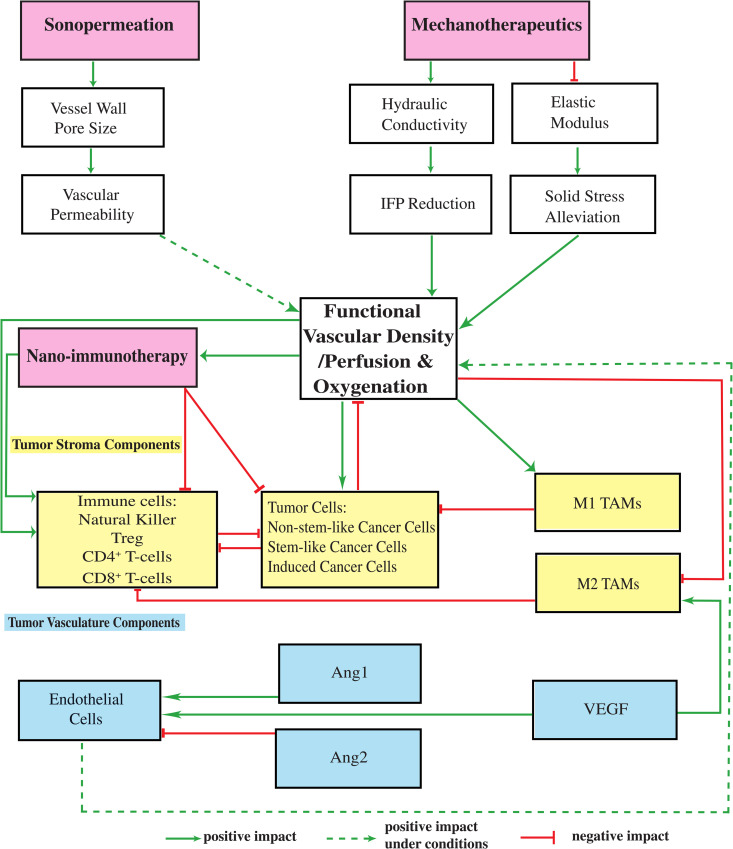
Schematic representation of the components of the mathematical model and their interrelations. The model incorporates diverse populations of cells (illustrated as yellow boxes), tumor angiogenic factors (illustrated as blue boxes), and various therapeutic modalities, each exerting distinct influences within the Tumor Microenvironment (illustrated as pink boxes). This detailed illustration explains the interactions amongst each individual model component, as well as the various potential combinations of these model components, thereby revealing the effects that these interactions have on functional vascular density/perfusion, and oxygenation levels within the examined system. These effects may be classified as positive, negative, or even positive under conditions.

Tumor progression within the host tissue is formulated in a continuum mechanics framework using the multiplicative decomposition of the deformation gradient tensor. The model is deterministic, and we solve the quasi-static linear momentum balance to obtain the tumor’s equilibrium configuration at each time point [27,50–55]. A detailed presentation of the model’s equations, assumptions, and foundational principles can be found in the S1 Text. A schematic representation illustrating the various components of the tumor, which have been incorporated into the mathematical model alongside their interrelations, is presented in Fig 1.

The model accounts for the intricate interactions among various components (Fig 1) that are known to play pivotal role in the multifaceted process of tumor progression, such as diverse populations of i) tumor cells: non-stem-like cancer cells (CCs), stem-like cancer cells (SCCs), and treatment-induced cancer cells (ICCs) [52], ii) immune cells: NK cells, CD8^+^ T-cells, CD4^+^ T-cells, regulatory T-cells (Treg) and Tumor Associated Macrophages (TAMs), iii) components of the tumor vasculature: Endothelial Cells (ECs), Angiopoietins (Ang), and the Vascular Endothelial Growth Factor (VEGF). The model further accounts for the degree of tumor perfusion, oxygenation and drug delivery (i.e., nano-immunotherapy). Angiopoietin-1 (Ang1) and Angiopoietin-2 (Ang2) are ligands of the Tie2 receptor that regulate blood vessel stability. Ang1 stabilizes vessels by promoting endothelial integrity and pericyte support, leading to mature, non-leaky vasculature. Ang2 acts as a context-dependent antagonist of Ang1, promoting vessel destabilization and angiogenesis when VEGF is present [56].

Sonopermeation facilitates an increase in the vessel wall pore size, thereby enhancing vascular permeability, which might augment functional vascular density [38]. The influence of mechanotherapeutics is essential in impacting both the fluid phase and the solid phase of the TME. With respect to the fluid phase, the application of mechanotherapeutics results in a significant elevation of the tumor hydraulic conductivity, which subsequently leads to a decrease in the Interstitial Fluid Pressure (IFP); this decrease, in turn, contributes to an increase in perfusion [6,28]. As far as the solid phase is concerned, mechanotherapeutics play a critical role in inducing a reduction in the elastic modulus, which ultimately results in the alleviation of solid stress; this alleviation of stress promotes vessel decompression, leading to an increase in functional vascular density/perfusion [6,28,29].

An improvement in functional vascular density enhances the efficacy of nano-immunotherapy, which consequently leads to a more effective suppression of non-stem-like cancer cells, stem-like cancer cells and induced cancer cells [6]. The enhanced concentration levels associated with nanotherapy increase the immunogenic cell death [57,58] and lead to an improvement in the efficacy of immunotherapy [19,50], which in turn results in an elevated ratio of CD4^+^/ CD8^+^ T-cells [59]. Through the synergistic effects of nano-immunotherapy, there is a promotion in the recruitment of effector CD8^+^ T-cells, while simultaneously there is a marked reduction in the frequency of regulatory T cells (Tregs), thus fostering a more effective anti-tumor immune response [60]. Furthermore, an increase in the oxygenation not only reinforces the populations of tumor and immune cells but also facilitates the polarization of tumor-associated macrophages (TAMs) from an immune-suppressive M2-phenotype to an immune-activating M1-phenotype [51,61,62].

However, rapid tumor-cell proliferation can counteract these benefits by re-establishing mechanical and immunological barriers. The proliferation of tumor cells leads to significant compression of the surrounding vasculature, which in turn results in a decrease in perfusion and a consequent inactivation of immune cells within the TME [11,63]. The enhancement of immune cell proliferation boosts the efficacy of tumor cell eradication. M1-like TAMs exert a substantial tumoricidal effect on tumor cells, whereas the M2-like TAMs inhibit the activity of immune effector cells, resulting in the inactivation of immune responses and promoting an immunosuppressive environment [64,65].

Regarding the tumor vascular components: The process of angiogenesis, which is a fundamental mechanism crucial for the formation of new blood vessels, is triggered through the proliferation of endothelial cells that form the vessels, thereby augmenting the overall perfusion [52]. The elevated levels of VEGF is correlated with an increased number of M2-like TAMs, as well as an elevated proliferation rate of ECs [66]. Furthermore, the presence of high concentrations of Ang2 destabilize existing vessels by diminishing the production of ECs, a phenomenon that is inhibited by Ang1, which contributes in stabilizing vessels and promoting the production of endothelial cells [67,68].

### 2.2. Transport of drugs

#### 2.2.1. Transport of nanotherapy.

We postulated that the delivery of nanotherapy exists in three separate states: the nanoparticle carrier containing the chemotherapy (c_n_), the chemotherapeutic agent free to travel in the interstitial space (c_f_) and the chemotherapeutic substance internalized by cells (c_int_) [69]. Therefore, the transport of the drug within the interstitial space can be represented as [70]:


$$\frac{\partial c_{n}}{\partial t}+\nabla \cdot \left(c_{n}vf\right)=D_{n}\nabla 2\;c_{n}+Q_{sta}-k_{el}c_{n}\mathrm{\backslash ]}$$ (1)

$$\frac{\partial c_{f}}{\partial t}+\nabla \cdot \left(c_{f}vf\right)=D_{f}\nabla 2\;c_{f}+\alpha \;k_{el}c_{n}-k_{int}c_{f}\mathrm{\backslash ]}$$ (2)

$$\frac{\partial c_{int}}{\partial t}+\nabla \cdot \left(c_{int}vs\right)=k_{int}c_{f}-k_{\deg}c_{int}\mathrm{\backslash ]}$$ (3)

where D_n_ and D_f_ denote the diffusion coefficients of the nanoparticle and chemotherapy within the tumor interstitial space, respectively. The variables k_el_, k_int_ and k_deg_ represent the rate constants for the chemotherapy release, internalization of the drug by the cells, and the degradation rate of the chemotherapeutic agent. Furthermore, α is the number of chemotherapy molecules contained in the nanocarrier and v^f^ and v^s^ are the velocities of the fluid and solid phase, respectively. More information regarding v^f^ and v^s^ is detailed in the S1 Text, Equations (S18-S26). In the present study, the specific type of nanotherapy employed is Doxil. Doxil (also known as Caelyx) is a liposomal formulation of the chemotherapy drug doxorubicin, developed to improve delivery to tumors and reduce toxicity. The drug is encapsulated in polyethylene glycol (PEG)-coated liposomes, which extend circulation time, enhance tumor accumulation via leaky vessels, and minimize side effects—especially cardiotoxicity [71]. The term Q_sta_ on the right side of Eq. (1) denotes the transport of the nanocarrier across the tumor vessel wall and it is defined by Starling’s approximation as [70]:


$$Q_{sta}=P_{er}S_{V}(C_{iv}-c_{n})+L_{p}S_{V}(P_{V}-p_{i})(1-\sigma_{f})C_{iv}\mathrm{\backslash ]}$$ (4)

where P_er_ is the vascular permeability, S_v_ the vascular density and p_i_ is the interstitial fluid pressure. Also, L_p_ denotes the hydraulic conductivity of the vessel wall, C_iv_ = exp(-(t-t_0_)/k_d_) represents the vascular concentration of the administered drug, which is indicative of a bolus injection, with t_0_ being the time of drug administration and k_d_ denoting the blood circulation decay, while σ_f_ is the reflection coefficient. The vascular conductivity L_p_ is determined as a function of the vessel wall pore radius and the parameters P_er_ and σ_f_ are considered as a function of the ratio of the radius of the drug to the radius of the pores of the vessel wall [72]. The right-hand side of Eq. (4) accounts for two key mechanisms governing transvascular transport. The first term P_er_ S_v_ (C_iv_ - c_n_) represents transvascular diffusion, driven by concentration gradients across the vessel wall, while the second term L_p_ S_v_ (P_v_ - p_i_)(1 - σ_f_) C_iv_ captures transvascular convection, driven by pressure differences between the vasculature and the interstitial space. Together, these mechanisms describe the extravasation of nanocarriers from the blood vessels into the tumor tissue. A detailed description of the methodology and calculations employed in deriving these values can be found within the S1 Text.

#### 2.2.2. Transport of immune checkpoint antibodies.

Immunotherapy is integrated into our mathematical framework through the incorporation of immune checkpoint antibodies, which, in certain therapeutic modalities, can be concurrently utilized to enhance treatment efficacy [52]. In the model, the effect of anti-PD-1 immune checkpoint inhibition is conceptualized as an augmentation in the source term of CD8^+^ T-cells, i.e., the term σ__Τ_8_ [52]. The term σ_T8_ is described in Equation S13 of the S1 Text. This equation outlines the interactions involving CD8 ⁺ T-cells within the tumor microenvironment. Furthermore, the anti-PD-1 antibody is also integrated in our computational model as a free pharmacological agent $c_{f_{i}}$, and this incorporation is illustrated through the mathematical representation provided in Equation (5).


$$\frac{\partial c_{f_{i}}}{\partial t}+\nabla \cdot \left(c_{f_{i}}v^{f}\right)=D_{f_{i}}\nabla^{2}c_{f_{i}}+Q_{sta_{i}}-k_{\deg_{i}}c_{fi}\mathrm{\backslash ]}$$ (5)

where ${D_{f}}_{i}\backslash )$ denotes the diffusion coefficient of the immune checkpoint antibody within the tumor interstitial space, ${k_{\deg}}_{i}\backslash )$ represents the rate constant for the degradation of the anti-PD-1 antibody and v^f^ is the interstitial fluid velocity. More information regarding v^f^ is given by S1 Text, Equation (S23). The term ${Q_{sta}}_{i}$ on the right side of Eq. (5) denotes the transport of the immune checkpoint antibody across the tumor vessel wall and it is defined by Starling’s approximation. It is crucial to recognize that Equation (4), is similarly applied in the context of the transport dynamics concerning the anti-PD-1 antibody, which is capable of moving through the interstitial space.

## 3. Materials and methods II

### 3.1. Modeling the effects of mechanotherapeutic ketotifen

Within the framework of this mathematical model, the incorporation of the mechanotherapeutic ketotifen has been integrated to facilitate the alleviation of stress within solid tumors, thereby leading to a consequential enhancement in the overall efficacy of nano-immunotherapy [6]. The effects associated with ketotifen are a reduction in tumor stiffness, coupled with a notable increase in vascular perfusion, thus enhancing the overall functionality of blood vessels. Experimental data indicate that, three days upon the administration of ketotifen, there is a significant reduction in tumor stiffness by approximately 50% [6,28,29]. Furthermore, ketotifen effectively reduces interstitial fluid pressure, facilitates improved tumor perfusion and markedly augments the efficacy of drug delivery [20]. In our model, we simulate the effects of ketotifen by reducing the tumor’s shear modulus and increasing the hydraulic conductivity, which in turn leads to a significant reduction in interstitial fluid pressure and increase in functional vascular density.

More specifically, upon the administration of ketotifen, there is a linear decrease in both the shear modulus and the bulk modulus within the tumor tissue in half, which can be quantitatively assessed in relation to the baseline values of these mechanical parameters prior to treatment [28]. Concurrently, the hydraulic conductivity exhibits a linear increase of two orders of magnitude when compared with the initial value of this parameter in the absence of ketotifen intervention. The precise numerical values that are relevant to hydraulic conductivity, *k*_*th*_, shear modulus, *μ*, and bulk modulus, *k*, concerning the host tissue, the tumor tissue and the tumor tissue with the effect of ketotifen are illustrated in S1 Table.

### 3.2. Incorporation of sonopermeation

Regarding the phenomenon of sonopermeation, the effect of acoustic pressure on vessel wall pores size has been added in the mathematical model. The process of sonopermeation is known to induce an increase in the size of the pores within the vessel wall, thereby augmenting vascular permeability, which in turn leads to a significant improvement in the functional density of the vascular network. Specifically, it has been demonstrated that sonopermeation possesses the capability to enlarge cell pores, with average dimensions that can vary significantly from 100 nanometers to 1.25 micrometers, and it is noteworthy that the generation of larger sonopermeation pores is positively correlated with an increase in acoustic pressure or an extension of treatment duration of the sonopermeation [73]. By correlating the empirical observations obtained from the aforementioned experimental study with the parameters delineated within our mathematical model, particularly in relation to the influence of acoustic pressure on the size of pores within the vessel wall, we have developed the following second-order polynomial function:


$$r_{o}=-14977.9087*{\text{p}}_{\text{ac}}^{2}+8208.3947*p_{ac}-69.0722,$$ (6)

The mathematical expression outlined in Equation (6) describes the effect of acoustic pressure, denoted as p_ac_, on the radius of the pore size within the vessel wall, represented by the variable r_o_, when sonopermeation is applied. The fitting curve and the corresponding data are provided in the S6 Fig.

The integration of the acoustic pressure into our mathematical model is accomplished via the subsequent relationship:


$$p_{ac}=MI*\sqrt{fr}\mathrm{\backslash ]}$$ (7)

The acoustic pressure, p_ac_, is quantitatively characterized by the mathematical relationship in which the Mechanical Index of the transducer, is multiplied by the square root of the frequency used for sonopermeation, fr, of the wave that has been transmitted [74,75]. The selection of values of mechanical index and frequency is based on previous clinical and experimental studies [6,38,39,41,76]. Specifically, in our previous study [6], we identified the optimal parameters of mechanical index (MI) - in the range of 0.2-0.6 - using a clinical ultrasound device with the C5-1 probe and frequency 2.2 MHz, which is employed in clinical trials [41,76]. Values of frequency and mechanical index are given in S1 Table. Importantly, in Equations 6 and 7, we neglect any attenuation and scattering effects due to the propagation of ultrasound waves and interaction with tissues and thus, the acoustic pressure remains constant and distributed uniformly within the tissue [47,48].

### 3.3. Solution methodology

To effectively model tumor growth, it is presumed that the tumor has a spherical configuration, surrounded by a normal tissue of cubic shape. The cubic host domain, which serves as the spatial environment for the tumor growth, is designed to be two orders of magnitude larger, thereby reducing any potential boundary effects that could interfere with the progression of the tumor. Due to symmetry present in the system under investigation, it is deemed sufficient to consider only one eighth of the entire system for analysis. The clarification of the boundary conditions that have been incorporated in this study is depicted in S1 Fig. In particular, the boundary conditions relevant to the conservation of both the stress and displacement fields, along with the concentration levels of oxygen as well as the immune-nanotherapeutic agents at the interface between the tumor tissue and the adjacent healthy tissue, are applied automatically by the software.

The system of equations that comprise the mathematical model was solved using the commercial finite elements software COMSOL Multiphysics (COMSOL, Inc., Burlington, MA, USA), using the Solid Mechanics, Transport of Diluted Species, Convection-Diffusion Equation and Domain ODEs and DAEs Physics. The computational domain is composed of 6015 finite elements and 51628 degrees of freedom; furthermore, the time-dependent solver that is employed to derive the solutions to the equations governing this model is the PARDISO algorithm. The finite element implementation employs Lagrange shape functions with quadratic element order. The time integration is performed using the Backward Differentiation Formula (BDF) within PARDISO, with a maximum time step size of 0.25 days. The time-stepping strategy is set to “Free,” allowing the solver to adaptively select time steps within this constraint. Due to the high nonlinearity of the model, the system of equations is linearized at each time step using a Newton–Raphson method, with a maximum of 4 iterations per time step and a tolerance factor of 1.

## 4. Results

### 4.1. Comparative analysis of mathematical model predictions with experimental data

To evaluate the robustness of our mathematical framework and to justify the parameter values employed within the model, we conducted a validation analysis between model predictions with published experimental data [6]. *In vivo* experiments on murine sarcoma models were conducted to determine optimal conditions for combining mechanotherapeutics and ultrasound sonopermeation, in order to improve perfusion and nano-immunotherapy effectiveness [6]. The findings derived from the experimental study revealed that the incorporation of the anti-histamine ketotifen as a mechanotherapeutic with sonopermeation reduced mechanical forces by lowering collagen and hyaluronan levels by 50%, thus reshaping the tumor microenvironment. The combined effects of ketotifen and sonopermeation not only increased tumor perfusion six times but also improved drug delivery. Consequently, the antitumor effectiveness of the Doxil nanomedicine as well as anti-PD-1 immunotherapy was significantly enhanced.

**Fig 2:**
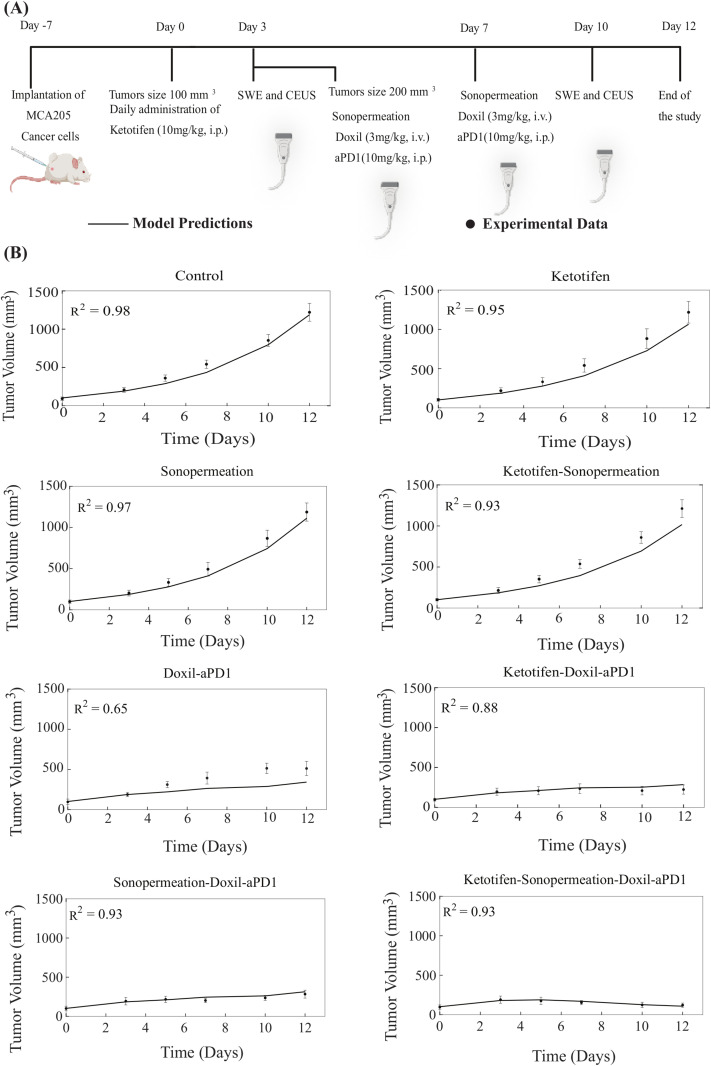
Comparison of model predictions with experimental data of tumor growth for MCA205 fibrosarcoma tumors. **(A)** Experimental treatment protocol followed for MCA205 fibrosarcoma tumors and simulated by the model. Created in BioRender.com. **(B)** Tumor volume growth rates of murine fibrosarcoma cancer cells (dots) and mathematical model predictions (solid lines) for each treatment group. For each case - control, ketotifen, sonopermeation, ketotifen-sonopermeation, Doxil-aPD1, ketotifen-Doxil-aPD1, sonopermeation-Doxil-aPD1 and ketotifen-sonopermeation-Doxil-aPD1- the R-Squared (R^2^) value has been calculated and depicts the accuracy of mathematical model validations for tumor growth in comparison with experimental findings. aPD1 denotes for anti-PD1 antibody.

**Fig 3:**
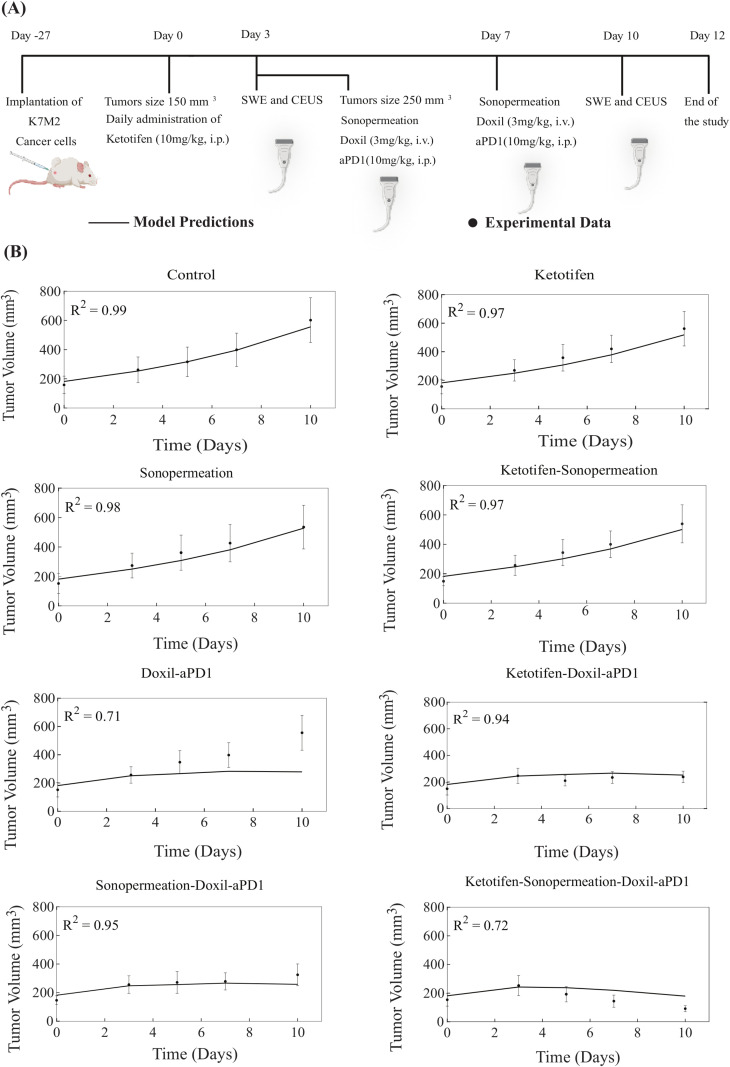
Comparison of model predictions with experimental data of tumor growth for K7M2 osteosarcoma tumors. **(A)** The experimental treatment protocol implemented for K7M2 osteosarcoma tumors and simulated by the model. Created with BioRender.com. **(B)** The tumor volume growth of murine osteosarcoma cells (dots) along with the predictions derived from mathematical modeling (solid lines) for each treatment group. For each case - control, ketotifen, sonopermeation, ketotifen-sonopermeation, Doxil-aPD1, ketotifen-Doxil-aPD1, sonopermeation-Doxil-aPD1 and ketotifen-sonopermeation-Doxil-aPD1- the R-Squared (R^2^) value has been calculated and depicts the accuracy of mathematical model validations for tumor growth in comparison with experimental findings. aPD1 denotes for anti-PD1 antibody.

The therapeutic regimen implemented through mathematical modeling was analogous to the experimental protocol utilized for MCA205 fibrosarcoma and K7M2 osteosarcoma tumors (Figs 2A and 3A). For the experimental protocol, mice were randomized into the following groups once tumors reached an average size of 100 mm^3^ (n = 8–10 per group): Control group, ketotifen (10 mg/kg, i.p.), sonopermeation, ketotifen + sonopermeation, Doxil (3 mg/kg, i.v.) + immune checkpoint inhibitor (ICI; anti-PD-1, 10 mg/kg, i.p.), ketotifen + Doxil + ICI, sonopermeation + Doxil + ICI, and ketotifen + sonopermeation + Doxil + ICI. Daily administration of ketotifen began once tumors reached approximately 100 mm^3^. After three days of ketotifen treatment—by which time tumors had grown to an average volume of 200 mm^3^—mice were subjected to sonopermeation. One hour later, Doxil and ICI were administered to enhance therapeutic efficacy. Sonopermeation was then employed to synergistically augment the antitumor efficacy of Doxil that has a size of 100 nm in diameter and the immune checkpoint antibody that was taken to have a size of 12 nm. The combined treatment of sonopermeation and nano-immunotherapy was subsequently repeated after four days [6]. The elastic modulus and perfused area were measured during the experimental procedure on specific days utilizing Shear Wave Elastography (SWE) and Contrast Enhanced Ultrasound (CEUS), respectively, whereas tumor volume was measured using a digital caliber. CEUS captures real-time perfusion dynamics in vivo, as illustrated by representative images of tumor perfusion reported in various experimental studies [6,77].

In order to carry out a comparison between the predictions generated by the model and the experimental data, all model parameters were set to baseline values derived independently from relevant studies (S1 Table). The only model parameter that underwent modification in order to adequately align the model’s predictions with the experimental findings [6] was the parameter denoted as *k*_*1*_, which serves to quantify the relationship between the proliferation of cancer cells and the concentration of oxygen (S1 Text, Equation S8). The value assigned to *k*_*1*_ was determined by fitting the tumor growth of the control/untreated group, and this value was kept the same across all comparisons of model predictions against the complete set of experimental data derived from all groups participating in the same study. For each sarcoma cancer type, *k*_*1*_ was varied to achieve alignment between the model predictions and the experimental results (S2 Table). Specifically, for each sarcoma cell line, we apply an optimization algorithm based on our previous studies [78,79] to compute the quality-of-fit metric (R^2^) between simulated and experimental tumor-volume measurements across all recorded time points, with R^2^ approaching 1 indicating a perfect fit. Since each cell line used in the experimental data exhibits a distinct growth profile, calibrating *k*_*1*_ for each type ensures that the model accurately reflects the biological variability in tumor growth rates.

The tumor volume estimations derived from mathematical modeling exhibit strong agreement with the experimental data, as monotherapies—namely control solution, ketotifen, sonopermeation, and the combination of ketotifen with sonopermeation—demonstrated no antitumor effects in terms of tumor volume reduction relative to the control group. Conversely, a notable reduction in overall tumor volume was observed for the combinatorial treatment. Our model substantiates that the combination of ketotifen and sonopermeation with nano-immunotherapy significantly improved therapeutic outcomes and particularly suppressed tumor growth (Figs 2B, 3B, S3 and S4). A more pronounced inhibition in tumor growth for both MCA205 and K7M2 sarcomas was recorded in the treatment modality where ketotifen, sonopermeation, Doxil, and anti-PD1 antibody were combined, resulting in effective tumor volume reduction. The predictive capacity of the mathematical model, which aligns closely with experimental observations, can be evaluated using the R-Squared (R^2^) statistic, which measures the precision of the correlation between experimental data and model forecasts, ranging from 0 to 1, with enhanced precision indicated by R^2^ values approaching 1.

**Fig 4:**
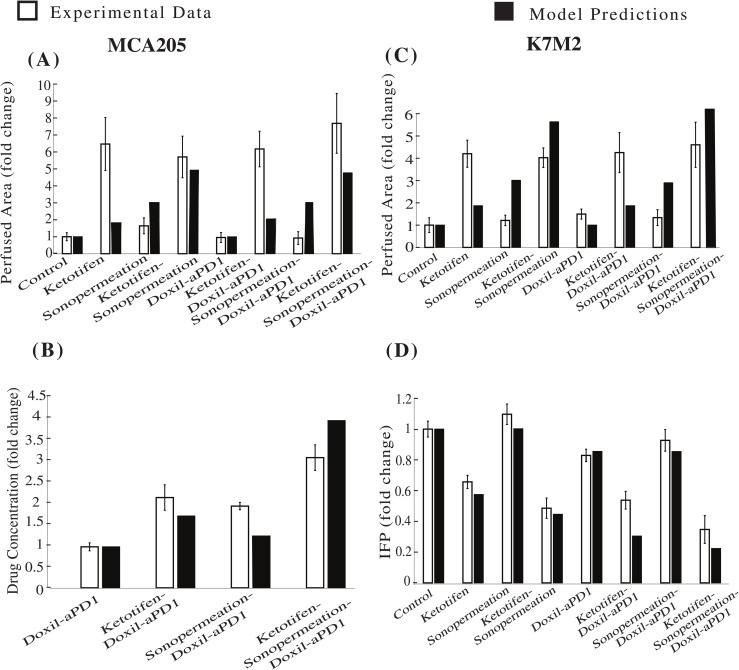
Comparison of model predictions in conjunction with experimental data [6] at Day 10. The horizontal axis delineates the various treatment groups that were included in the experimental investigations: control, ketotifen, sonopermeation, ketotifen-sonopermeation, Doxil-aPD1, ketotifen-Doxil-aPD1, sonopermeation-Doxil-aPD1, ketotifen-sonopermeation-Doxil-aPD1. The vertical axis (y) for each instance varies between **(A)** Perfused Area and **(B)** Drug Concentration for MCA205 fibrosarcoma, **(C)** Perfused Area and **(D)** Interstitial Fluid Pressure (IFP) for K7M2 osteosarcoma.

To elucidate the comparative analysis between the model and experimental data, we further compared model predictions with experimental measurements of perfused area using contrast enhanced ultrasound and drug concentration measured with fluorescence imaging and with IFP measurements [6] (Figs 4 and S5). For clarity, “drug concentration” is used to denote the intracellular (internalized) chemotherapeutic agent *(c*_*int*_*)*. To correlate the parameters of the model and the actual measurements obtained from the experiments, the values corresponding to the various measured parameters are presented in relation to the values of the control group, specifically represented as a fold change. The results shown in Fig 4 were obtained on Day 10, which corresponds to the measurement time points indicated in the experimental protocols in Figs 2A and 3A. It is noteworthy that model predictions are in good agreement with the experimental data of the perfused area, the levels of drug concentration and the IFP as illustrated in Fig 4. In situations where ketotifen is administered in conjunction with sonopermeation and concurrent administration of ketotifen-sonopermeation-Doxil-aPD1, it is observed that there is a significant enhancement in the perfused area, which in turn leads to an improvement in the functional vascular density Fig 4A and 4C. Moreover, by comparing the concentration levels of the drug with the various experimental groups that have been administered Doxil and aPD1, the predictions generated by our model exhibit a good level of agreement with the experimental data, illustrating that the synergistic effects resulting from the combination of ketotifen and sonopermeation indeed contribute to an enhancement in drug delivery for MCA205 fibrosarcoma tumors (Fig 4B). Last but not least, the combination of both ketotifen and sonopermeation prior to the injection of nano-immunotherapy results in a substantial decrease in interstitial fluid pressure (IFP) when compared to the group that received only ketotifen or the group that received both ketotifen and nano-immunotherapy in K7M2 osteosarcoma tumors (Fig 4D).

Our mechanistic model enables us to interpret the biological processes underlying the observed changes in tumor volume, perfused area, drug concentration and IFP across different treatment groups (Figs 2–4). In Figs 2 and 3, the changes in tumor volume for MCA205 and K7M2 sarcoma models can be explained by the model’s integration of mechanical, vascular, and immune dynamics. For instance, monotherapies such as ketotifen or sonopermeation alone do not lead to any tumor shrinkage, consistent with their predicted impact on the TME (e.g., relieving mechanical stress or enhancing vascular permeability without directly killing cancer cells). However, when combined with nano-immunotherapy, these TME interventions enhance drug delivery and immune cell activation because of enhancements in tumor perfusion and oxygenation, which in turn accelerates tumor cell death and suppresses tumor growth. In Fig 4, in cases where ketotifen is employed, the model simulates a reduction in tumor stiffness and solid stress, which results in vascular decompression and improved vessel functionality. The increase in functional vascular density is reflected in both model and experimental perfused area data. When sonopermeation is added, the model simulates a further increase in vascular permeability, which enhances nanoparticle and antibody transport across vessel walls. This explains the marked improvement in drug concentration observed in groups treated with combination of ketotifen and sonopermeation. In terms of IFP, the model captures how ketotifen-induced increases in hydraulic conductivity lead to pressure relief in the interstitial space because the fluid can more effectively escape from the tumor to the surrounding normal tissue. IFP reductions can also establish a pressure gradient across the vessel walls, allowing drug extravasation through convection, which is a dominant transport mechanism for nanoparticles and antibodies compared to diffusion. Overall, the model provides a framework linking TME modulation to therapeutic efficacy, explaining why combinatorial therapies cause synergistic effects on tumor growth, perfusion, drug concentration, and IFP.

### 4.2. Parametric analysis of essential variables for sonopermeation

The goal of the parametric analysis is to elucidate the specific values of distinct parameters that exert the most significant influence on the phenomenon of sonopermeation and consequently, the resultant therapeutic outcomes that can be achieved through this technique.

Upon the application of sonopermeation, there is a marked enhancement in the pore size, or permeability, of the vessels walls associated with tumor tissues, leading to greater drug infiltration. In order to facilitate a controlled and effective delivery of the therapeutic agent without incurring any detrimental damage to surrounding tissues, it is imperative to meticulously regulate the parameters associated with ultrasound application. In the context of sonopermeation, the ultrasound is typically applied in a pulsed manner to reduce tissue damage from excessive heating and enable microbubble inflow during pulse intervals, especially when bubble destruction is likely. The sinusoidal ultrasound wave is characterized by parameters such as velocity, wavelength, frequency, pressure amplitude, pulse length (burst duration), pulse repetition frequency (PRF), exposure time (duty cycle), treatment duration, and post-sonopermeation effects [34,80–82]. The parameters commonly used to induce sonopermeation and promote drug delivery exhibit considerable variability across different studies, encompassing frequency ranges from 0.5 to 3 MHz, pressure levels between 0.05 to 2 MPa, and total treatment durations that can span from seconds to several hours [38,81,83–89]. Additionally, we employed low Mechanical Index values within the range of 0.05 to 0.3 to induce stable cavitation, avoiding inertial cavitation that can cause tissue damage [38]. Furthermore, the fundamental effect of sonopermeation persists for a duration ranging from 4 to 24 hours, and after the lapse of 24 hours, it reveals no substantial effect [90,91].

In view of the previously mentioned parameters, we opted to integrate and simulate within our mathematical model the frequency employed for sonopermeation, the acoustic pressure that we correlate with the mechanical index (MI) through the Equation (7) and the duration of the effect of sonopermeation treatment. To perform parametric analysis, we varied values of frequency and mechanical index within the range given previously and check treatment efficacy for different time points that sonopermeation effect occurs.

**Fig 5:**
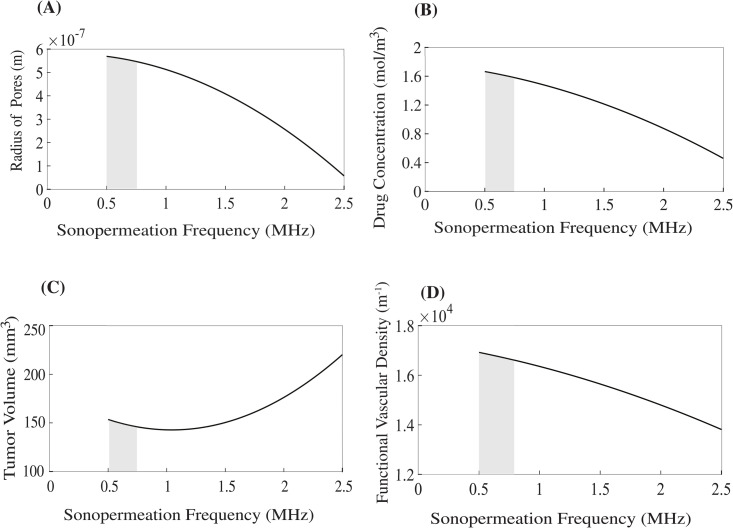
Impact of sonopermeation frequency on the following parameters: (A) pore radius (m), (B) drug concentration (mol/m^3^), (C) tumor volume (mm^3^) and (D) functional vascular density (m^-1^). The values corresponding to the various model parameters were computed at a position that is equidistantly located between the central region of the tumor and its peripheral boundaries. Grey regions depict the optimal range of frequencies. Optimal area was identified using the top-K percent criterion (i.e., k = 5%) by selecting values in the x axis (i.e., frequency) associated with values in the y axis within 95-105% of minimum tumor volume (Panel C). This frequency range was the same for the rest of the panels.

Fig 5 depicts the impact exerted by the frequency of sonopermeation, keeping the mechanical index constant (MI = 0.3), on various critical parameters, including the pore radius, tumor volume, functional vascular density and the concentration of the administered drug. A detailed examination reveals that when the sonopermeation frequency lies within a specific range—particularly between 0.5 and 0.8 MHz— the results indicate optimal outcomes across all the aforementioned parameters represented in this figure. The greatest effects are found for a frequency equals to 0.5 MHz. The optimal frequency range is highlighted with a grey region within the figure.

**Fig 6:**
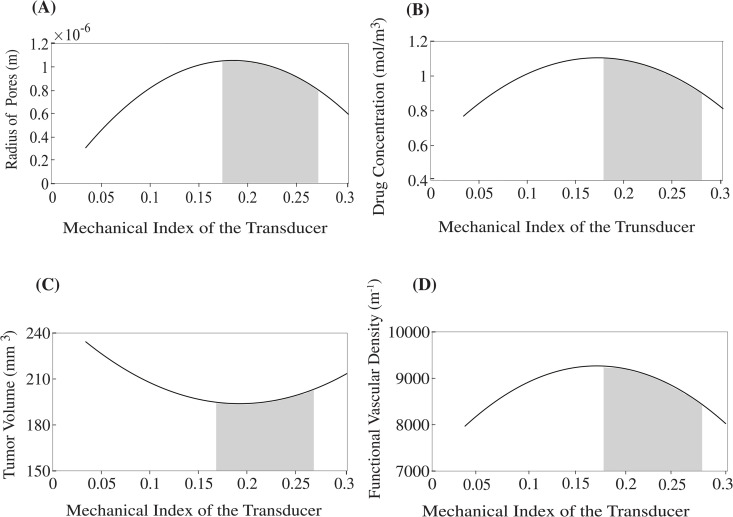
Impact of mechanical index of the transducer on the following parameters: (A) pore radius (m), (B) drug concentration (mol/m^3^), (C) tumor volume (mm^3^) and (D) functional vascular density (m^-1^). The values corresponding to the various model parameters were computed at a position that is equidistantly located between the central region of the tumor and its peripheral boundaries. The grey region depicts the optimal range of the mechanical index. Optimal area was identified using top-K percent criterion as described previously in the caption of Fig 5.

In Fig 6, we examined the impact of the mechanical index of the transducer, keeping the frequency constant (fr = 2.2 MHz), on the pore radius, tumor volume, functional vascular density, and the concentration of the relevant drug. The most favorable results become apparent when the mechanical index is approximately 0.17 with an optimal range between 0.17-0.27, as these values correlate positively with all the parameters that are illustrated. The optimal mechanical index range is marked with grey within the figure to aid visual interpretation. In conclusion, it can be stated that when the frequency of sonopermeation (fr) approaches 0.5 MHz and the mechanical index is around the value of 0.17, the corresponding acoustic pressure (p_ac_) is found to be 0.24 MPa and 0.25 MPa, respectively. Importantly, these values are in accordance with the values used in clinical trials [41,76].

**Fig 7:**
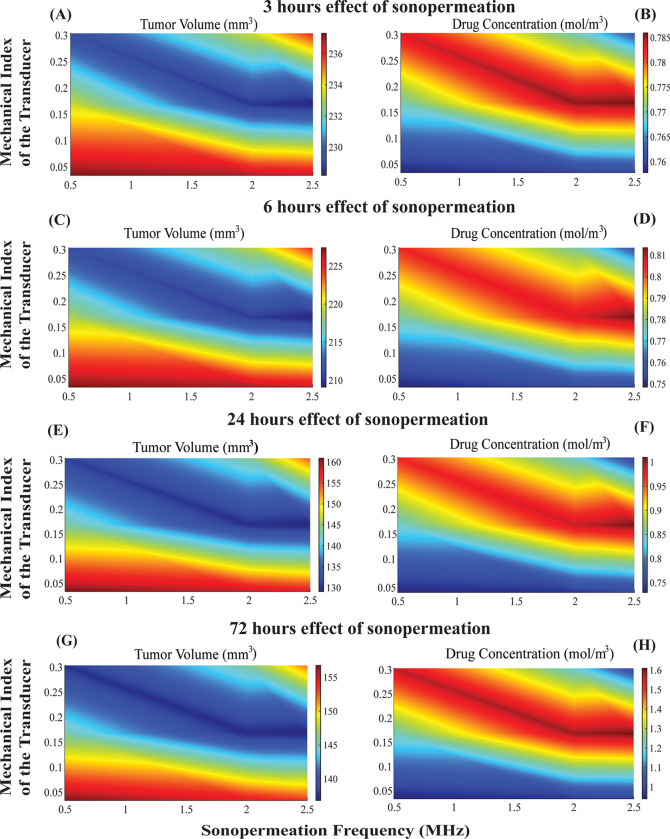
The influence of varying sonopermeation frequencies, in conjunction with diverse values of the mechanical index of the transducer utilized for the application of sonopermeation, on the synergistic effects of combined therapy is analyzed. Phase diagrams depict the impact of treatments on tumor volume **(A)**, **(C)**, **(E)**, and **(G)** as well as drug concentration **(B)**, **(D)**, **(F)**, and **(H)** across different time frames of the effect of sonopermeation. Specifically, **(A)** and **(B)** correspond to a 3-hours **(C)** and **(D)** to a 6-hours, **(E)** and **(F)** to a 24-hours effect of sonopermeation and **(G)** and **(H)** to a 72-hours effect of sonopermeation. The circles on the plots depict the regions where treatment is optimized. Optimal area was identified using top-K percent criterion as described in the caption of Fig 5.

The optimal regions presented in Figs 5–7 were determined using a top-K selection method, which involves identifying the parameter combinations that yield tumor volumes within ±5% of the minimum value observed for each treatment scenario. This range defines the optimal region for tumor volume. The same parameter combinations are then used to extract the corresponding values for other key model outputs, including pore radius, drug concentration, and functional vascular density. For example, in Fig 5C, the optimal tumor volume is 146.0 mm^3^ at a frequency of 0.5 MHz. Applying a ± 5% margin results in an upper bound of 153.7 mm^3^, observed at a frequency of 0.8 MHz. Therefore, the optimal region in this case corresponds to the frequency range of 0.5–0.8 MHz. Additionally, the model results shown in Figs 5 and 6 were calculated 1 day after treatment, to remain consistent with experimental findings indicating that the therapeutic effects of sonopermeation persist for approximately 24 hours [90,91].

Subsequently, we determined the optimal combination of frequency and mechanical index values that maximized treatment effectiveness. As illustrated in Fig 7, for a given frequency between 0.5 to 2.5 MHz and values of mechanical index between 0.17 to 0.3, the antitumor efficacy is enhanced which agrees with the conclusions reached from Figs 5 and 6. However, it becomes clear that the optimal combination for minimizing tumor volume and maximizing drug concentration, is attained when the frequency is defined in the vicinity of 2.5 MHz, the mechanical index is taken in the vicinity of 0.17, and consequently the acoustic pressure is calculated to be 0.27 MPa. Thus, it is evident that the most favorable outcomes manifest when the acoustic pressure is maintained within the critical range of 0.24 to 0.27 MPa, as this condition appears to optimize the critical parameters being analyzed. The maintenance of acoustic pressure within the specific and critical parameters ranging from 0.24 to 0.27 MPa yields the most favorable and effective outcomes, as this particular condition seems to enhance the concentration of the administered pharmaceutical agents and thus, antitumor effects. These observations are in accordance with the values employed for sonopermeation in a clinical trial for patients with pancreatic cancer where the mechanical index was equal to 0.2 and the pressure to 0.27 MPa [41]. Additionally, these values are related to ISPTA (Spatial-Peak Temporal-Average Intensity) close to 1 W/cm^2^, which is within the safe and effective limits of Low-Intensity Ultrasound. It is important to note that the ISPTA value presented here is an approximate calculation. This estimation assumes that all acoustic energy generated by the transducer reaches the tumor without loss, which likely leads to an overestimation of the actual intensity within the tumor tissue. In practice, energy attenuation due to absorption, scattering, and reflection in overlying tissues can significantly reduce the intensity that reaches the tumor.

Extending the durations of the effect of sonopermeation from 24 hours to 72 hours leads to a further rise in intratumoral drug concentration (note the higher color-bar values in Fig 7H versus 7F), this additional accumulation does not translate into greater tumor shrinkage: the simulated tumor volumes at 72 h (Fig 7G) and 24 h (Fig 7E) are essentially indistinguishable. Thus, the therapeutic benefit of sonopermeation plateaus within the first 24 h, corroborating our experimental observation that its biological effect lasts predominantly for a maximum of 24 hours. As evidenced by the data presented in Fig 7E and 7F, one can observe a remarkable reduction in tumor growth that occurs alongside the elevated concentration of the drug, in addition to the notable increased in vascular density and the optimization of pore size. In Fig 7H the drug concentration is higher than in the Fig 7F but the tumor volume is approximately the same (Fig 7E and 7G).

**Fig 8:**
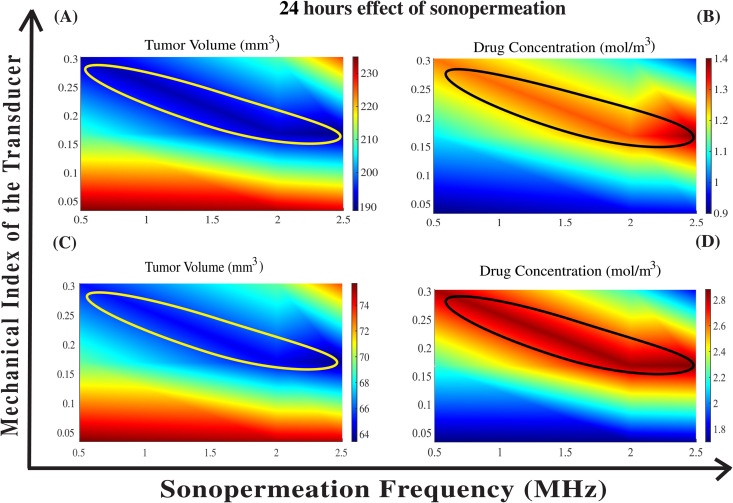
The impact of varying sonopermeation frequencies, in combination with different values of the mechanical index of the transducer applied for the application of sonopermeation, on the synergistic effects of the combined therapy. Phase diagrams illustrate the effects on tumor volume **(A, C)** and drug concentration **(B, D)** over a 24-hour sonopermeation treatment period. Specifically, panels **(A)** and **(B)** correspond to *k*_*1*_ = 0.62 day^−1^, while panels **(C)** and **(D)** correspond to *k*_*1*_ = 0.5 day^−1^. Circles on the diagrams indicate regions of optimal therapeutic performance, identified using the top-K percent criterion detailed in the Fig 5 caption.

In Fig 8, we examine the effect of the proliferation rate constant, *k*_*1*_, on the model predictions. We varied the frequency of sonopermeation in the range of 0.5 to 2.5 MHz, the mechanical index values between 0.17 and 0.3, and kept the effect of sonopermeation duration to 24 hours, as this represents the most realistic experimental scenario [90,91]. The cancer cell proliferation rate constant, *k*_*1*_, took values of 0.62 day^−1^ and 0.5 day^−1^, representing slightly higher and lower values compared to the baseline *k*_*1*_ = 0.56 day^-1^ employed in Fig 7. As shown in Fig 8A and 8B for *k*_*1*_ = 0.62 day^-1^ and Fig 8C and 8D for *k*_*1*_ = 0.5 day^-1^, the most effective combination for reducing tumor volume and increasing drug concentration consistently occurs at a frequency around 2.5 MHz and a mechanical index near 0.17, corresponding to an acoustic pressure of approximately 0.27 MPa. Therefore, in both cases-Fig 8A and 8B and Fig 8C and 8D-the optimal sonopermeation parameters for therapeutic benefit within the first 24 hours remain the same as those identified in Fig 7E and 7F for *k*_*1*_ = 0.56 day^-1^. It is important to note that even with small variations in *k*_*1*_, both tumor volume and drug concentration change significantly; however, the optimal combination of frequency and mechanical index for maximizing therapeutic benefit remains the same. These findings are consistent with the sonopermeation parameters used in a clinical trial, where the acoustic pressure was set to 0.27 MPa [41].

## 5. Discussion

In this study, we have formulated a mechanistic mathematical framework that integrates the synergistic application of mechanotherapeutics and sonopermeation, with the primary objective of realizing the highest possible efficacy of nano-immunotherapy in the treatment of cancer. The model we developed encompasses the diverse interactions that occur among various categories of cancer cells, immune cells, tumor associated macrophages, endothelial cells, tumor angiogenic factors and an array of different therapeutic modalities, each of which exerts its unique and distinct influences within the complex domain of the tumor microenvironment. Our model represents a significant advancement over prior research endeavors, as it incorporates the effects of sonopermeation alongside the mechanotherapeutic agent ketotifen, which is known for its therapeutic potential. The complexity of our model can be substantiated by the favorable alignment of the predictions generated by the mathematical model with the experimental results obtained from in vivo studies on two distinct sarcoma cell lines that exhibit varying growth rates, specifically one characterized by a rapid growth rate (the fibrosarcoma MCA205) and another that demonstrates a comparatively slower growth rate (the osteosarcoma K7M2). The experimental data we have collected provide compelling evidence that the mechano-modulation of the tumor microenvironment, achieved through the combined application of mechanotherapeutics and sonopermeation, can result in multiplicative synergistic effects that significantly enhance perfusion and improve overall therapeutic outcomes [6]. These observations are validated with a good degree of precision through our mathematical model. A good correlation was observed between the computational values produced by the model and the actual experimental measurements of critical quantities of interest, such as tumor volume, functional vascular density, interstitial fluid pressure and drug concentration which are essential to reinforce the overall accuracy and reliability of the mathematical framework employed. To achieve this good correlation between model predictions and experimental data we adjusted only a single parameter—*k*₁**, which governs the proliferation rate of cancer cells—while keeping all other parameters fixed. This approach aimed to test whether varying a single parameter could capture general treatment trends across diverse therapeutic conditions, thereby assessing the model’s robustness and generalizability without case-specific calibration. While this simplification may limit quantitative accuracy, the model successfully reproduced relative behaviors and response trends in most scenarios. The objective was to demonstrate that a unified modeling framework, can yield qualitatively consistent and mechanistically meaningful results with minimal parameter adjustment.

The parametric analysis, which examined a variety of parameters, provided optimal guidelines that are essential for the effective implementation of sonopermeation. These parameters include the mechanical index of the transducer utilized, the specific frequency at which sonopermeation is conducted, the acoustic pressure applied during the procedure, the duration over which the effect of sonopermeation is exerted and the rate of proliferation of cancer cells. More specifically, the analysis delineates a precise range of values for these critical parameters within which the efficacy of sonopermeation is markedly enhanced, thereby yielding the most favorable and effective results. Based on the parametric analysis, the optimal conditions for maximizing drug concentration and minimizing tumor volume are achieved when the frequency is set to 2.5 MHz, the mechanical index is approximately 0.17, and the corresponding acoustic pressure is calculated to be 0.27 MPa. Importantly, these optimal values are in accordance with values employed for sonopermeation in a clinical trial for patients with pancreatic cancer, highlighting the clinical relevance of the model [41]. For the parametric analysis, we selected Mechanical Index values lower than 0.7, which promotes stable cavitation, thereby enhancing permeability while minimizing the risk of inertial cavitation and tissue damage [38,92].

The manuscript reports results of a pilot project and thus, it has certain limitations especially in relation to the application of the effect of sonopermeation within the framework of our model. Sonopermeation could improve the overall efficacy of drug delivery by leveraging additional mechanisms that are not explicitly included in the current model framework. For instance, the interaction of acoustic waves and the resultant shear wave stresses, that are exerted upon endothelial cells by the action of microbubbles has been defined in accordance with prior studies [46,93,94]. It is worth mentioning that the application of shear stress has been shown to reduce apoptosis in endothelial cells, thereby enhancing functional vascular density [95]. Specifically, physiological levels of laminar shear stress exert a potent suppressive effect on endothelial cell (EC) apoptosis triggered by various stimuli, thereby supporting endothelial integrity and enhancing functional vascular density. For example, studies have shown that pulsatile flow with a significant forward direction protects ECs from H_2_O_2_-induced cell death, whereas reciprocal flow does not confer such protective effects [24]. Additionally, laminar shear stress has been reported to upregulate inhibitors of apoptosis proteins (IAP-1 and IAP-2) [25,26], although the precise mechanisms underlying this induction remain to be fully elucidated.

Furthermore, in our mathematical model, we do not model the propagation of ultrasound waves ignoring attenuation and scattering effects, but we employ the effects of sonopermeation on various components of the tumor microenvironment based on experimental observations. Additionally, to predict tumor growth the model does not capture the high-frequency oscillations and accelerations associated with ultrasound microsecond timescale which is a much lower timescale compared to that of tumor growth [47]. Also, the model does not account for the effect of sonopermeation in signaling pathways, such as phosphorylation of MAP-kinases (MAPK) [37], and activation p38, ERK, Akt and integrin receptors—focal adhesion kinase (FAK) [96–99]. In addition, the effect of sonopermeation on the extracellular matrix (ECM) is not yet clearly defined and warrants further investigation. Previous studies have reported that sonopermeation does not significantly alter key ECM components such as hyaluronan, collagen, or cancer-associated fibroblasts (CAFs) [6,39,77]. Due to the lack of a clearly defined biological mechanism linking sonopermeation to ECM remodeling, we have not included direct ECM alterations in our current modeling framework. However, emerging experimental evidence suggests that ultrasound exposure may indirectly remodel the ECM. For example, Li et al. [100] demonstrated that pulsed high-intensity focused ultrasound (HIFU) combined with microbubbles enhanced doxorubicin uptake in a pancreatic cancer mouse model, which coincided with visible disruption of collagen fibers that appeared disorganized and loosely packed. Similarly, another study [101] reported that pulsed high-intensity ultrasound reduced collagen content in a lung carcinoma model, leading to increased nanoparticle penetration. While these studies suggest that sonopermeation may induce ECM remodeling under specific conditions, the effect is not yet well understood at the mechanistic level and thus is not incorporated into our current model. We acknowledge this as a direction for future refinement and exploration.

In this study, we model the tumor tissue as a poroelastic medium [102,49], where the solid matrix is treated as purely elastic and interstitial fluid transport follows Darcy’s law. This assumption is justified by the temporal and spatial scales of interest, which focus on the slow, macroscale proliferation of cancer cells. While it is well-established that tumor tissues can exhibit viscous behavior—particularly at the microscale—we have chosen to omit viscoelastic effects in the present model in order to avoid excessive complexity. We further acknowledge the limitation of not having used spatially-resolved data to calibrate and validate the model [103–106]. Although a full global sensitivity analysis would be valuable for classifying the many parameters and guiding future model refinement, it would be extremely time-consuming and computationally intensive. Therefore, we performed a selective sensitivity analysis focusing on the parameters with the greatest influence on our model, namely the ultrasound frequency, mechanical index, duration of the sonopermeation effect, and the cancer-cell proliferation rate parameter *(k*_*1*_*)*. Finally, to maintain computational tractability and avoid excessive parameterization, we employ an idealized tumor geometry that cannot encompass the full spatial heterogeneity and mechanistic complexity of tumors or the tumor microenvironment, even though it retains the key biophysical interactions between the tumor and surrounding healthy tissue. These limitations are expected to affect model predictions only quantitatively, while the main conclusions derived from this study remain the same.

## Supporting information

S1 Text Description of the mathematical model. (DOCX)

S1 Table Parameter values applied within the model. (DOCX)

S2 Table The value of the parameter k_1_, which is employed in the process of fitting the mathematical model to the experimental data for each cancer cell line. (DOCX)

S3 Table The initial values of the variables used in the mathematical model at time t = 0 day. (DOCX)

S1 Fig The computational domain along with the specified boundary conditions that have been utilized for the current analysis of stress (σ), displacement (u), the concentration of oxygen (c_ox_), and the concentrations associated with the nanotherapeutic agent Doxil, specifically denoted as c_n_, c_fn_, and c_int_, in addition to the immunotherapeutic agent anti-PD-1, represented by c_f_. (TIF)

S2 Fig Flowchart summarizing the computational workflow. The diagram illustrates the overall computational framework developed to evaluate therapeutic outcomes achieved through mechanotherapy and sonopermeation in solid tumors. It outlines the key assumptions of the model and the main input parameters incorporated into the mathematical formulation. The flowchart also presents the governing equations underlying the mathematical model, along with the numerical methods employed for their solution. Finally, the workflow highlights the resulting output variables that are used to assess therapeutic efficacy.
(TIF)

S3 Fig Comparison of model predictions with experimental data of tumor growth for MCA205 fibrosarcoma tumors. In these simulations, the host tissue is assigned a Poisson’s ratio of ν = 0.49, while the tumor tissue is modeled as incompressible, with a Poisson’s ratio of ν = 0.499999. **(A)** Experimental treatment protocol followed for MCA205 fibrosarcoma tumors and simulated by the model. Created in BioRender.com. **(B)** Tumor volume growth rates of murine fibrosarcoma cancer cells (dots) and mathematical model predictions (solid lines) for each treatment group. For each case - control, ketotifen, sonopermeation, ketotifen-sonopermeation, Doxil-aPD1, ketotifen-Doxil-aPD1, sonopermeation-Doxil-aPD1 and ketotifen-sonopermeation-Doxil-aPD1- the R-Squared (R^2^) value has been calculated and depicts the accuracy of mathematical model validations for tumor growth in comparison with experimental findings. aPD1 denotes for anti-PD1 antibody. We note that increasing the values of the Poisson’s ratio of the tumor and host tissue does not affect qualitatively our results and the model can still provide a good fit to the experimental data.
(TIF)

S4 Fig Comparison of model predictions with experimental data of tumor growth for K7M2 osteosarcoma tumors. In these simulations, the host tissue is assigned a Poisson’s ratio of ν = 0.49, while the tumor tissue is modeled as incompressible, with a Poisson’s ratio of ν = 0.499999. **(A)** The experimental treatment protocol implemented for K7M2 osteosarcoma tumors and simulated by the model. Created with BioRender.com. **(B)** The tumor volume growth of murine osteosarcoma cells (dots) along with the predictions derived from mathematical modeling (solid lines) for each treatment group. For each case - control, ketotifen, sonopermeation, ketotifen-sonopermeation, Doxil-aPD1, ketotifen-Doxil-aPD1, sonopermeation-Doxil-aPD1 and ketotifen-sonopermeation-Doxil-aPD1- the R-Squared (R^2^) value has been calculated and depicts the accuracy of mathematical model validations for tumor growth in comparison with experimental findings. aPD1 denotes for anti-PD1 antibody. We note that increasing the values of the Poisson’s ratio of the tumor and host tissue does not affect qualitatively our results and the model can still provide a good fit to the experimental data.
(TIF)

S5 Fig Comparison of model predictions in conjunction with experimental data [6] for a specific time point. In these simulations, the host tissue is assigned a Poisson’s ratio of ν = 0.49, while the tumor tissue is modeled as incompressible, with a Poisson’s ratio of ν = 0.499999. The horizontal axis delineates the various treatment groups that were included in the experimental investigations: control, ketotifen, sonopermeation, ketotifen-sonopermeation, Doxil-aPD1, ketotifen-Doxil-aPD1, sonopermeation-Doxil-aPD1, ketotifen-sonopermeation-Doxil-aPD1. The vertical axis (y) for each instance varies between **(A)** Perfused Area and **(B)** Drug Concentration for MCA205 fibrosarcoma, **(C)** Perfused Area and **(D)** Interstitial Fluid Pressure (IFP) for K7M2 osteosarcoma. Again, we find that changing the values of the Poisson’s ratio for the tumor and host tissue can still provide a good fit to the experimental data. We note that increasing the values of the Poisson’s ratio of the tumor and host tissue does not affect qualitatively our results and the model can still provide a good fit to the experimental data.
(TIF)

S6 Fig Polynomial fitting of experimental data [73] relating acoustic pressure (MPa) to mean pore size (nm). The second-degree polynomial curve was fitted to the data points, and the resulting equation is shown in the plot.
(TIF)
