# Supplementary material for: Optimizing therapeutic outcomes with Mechanotherapy and Ultrasound Sonopermeation in solid tumors
Source: PLoS Comput Biol. 2025 Sep 23;21(9):e1012676. doi: 10.1371/journal.pcbi.1012676 (PMC12483211; doi:10.1371/journal.pcbi.1012676)
Supplement: S1 Text — (DOCX) [file pcbi.1012676.s001.docx]

Supporting Information

**Optimizing therapeutic outcomes with Mechanotherapy and Ultrasound Sonopermeation in solid tumors**

Marina Koutsi, Triantafyllos Stylianopoulos, Fotios Mpekris

**Description of the mathematical model**

**Kinematic principles underlying tumor growth**

Equations (S1-S3) and (S18-S26) establish the comprehensive framework for the biomechanical model of tumor growth, which is solved to simulate the progression of the tumor within normal/host tissue. The solutions to these equations facilitate the computation of interstitial fluid pressure, fluid velocity, and the stresses generated through mechanical interactions between the tumor and the host tissue. Interstitial fluid pressure and fluid velocity are incorporated into the drug transport equations (Equations 1-3, 4, in the main text), while the mechanical interactions between the tumor and the host tissue influence the tumor's growth rate. Equations (S4-S17) serve to clarify the balance law concerning the various cell types within the tumor to measure the growth stretch ratio, $\lambda_{g}$, which determines the rate of tumor proliferation. This growth stretch ratio is utilized within the general framework via Equations (S2, S3, and S25). All parameter values of this mathematical model are detailed in **Table S1**. Also, **Table S3** illustrates the initial values of the variables used in the mathematical model at time t=0 day.

This mathematical framework employs a robust systems-biology approach, which is meticulously designed to incorporate the intricate cellular and subcellular phenomena that occur within biological systems, specifically utilizing a continuum mechanics and finite elements model to thoroughly analyze the multifaceted dynamics of tumor growth.

In order to describe the kinematic behavior of the tumor, the multiplicative decomposition of the deformation gradient tensor is utilized. The tensor $F$ is divided into two distinct components: the tumor's growth, denoted as , and the elastic mechanical interaction, represented as [1-4]:

|  | (S1) |
| --- | --- |

Henceforth, all tensor representations will be rendered in **boldface** to facilitate their distinction from other terms. It is important to recognize that, given the assumptions inherent in the model and the specific problem under investigation, the deformation gradient tensor can be expressed as a combination of more than two independent components, which separately encompass growth, residual stress formation, or extracellular matrix (ECM) remodeling [4-6]. The growth component, denoted as , has been characterized as isotropic and homogeneous, representing a non-stress inducing deformation gradient tensor that represents the proliferation of cancer cells [7-10].

|  | (S2) |
| --- | --- |

where is the growth stretch ratio, which assesses the proliferation of cancer cells, while $I$ represents the second-order identity tensor, indicating that the proliferation associated with tumor growth occurs uniformly and isotropically. The elastic component, , accounts for the stress-inducing elastic interactions both within the tumor and with the surrounding host tissue. The elastic component of $F$ was derived directly from Equation (S1) as:

|  | (S3) |
| --- | --- |

**Calculation of the growth stretch ratio**

The rate of tumor growth is measured by taking into account both the concentration of oxygen and the proliferation of cancer cells [7, 8, 11-13]. The equation utilized is:

| , | (S4) |
| --- | --- |

where $T_{CCs}$ represents the population of non-stem-like cancer cells (CCs), $T_{SCCs}$denotes the stem-like cancer cells (SCCs) population, $T_{ICCs}$signifies the induced cancer cells (ICCs) population, $T_{tot}$constitutes the total density of tumor cells as determined by the summation of the aforementioned three populations. The $S_{CCs}$, $S_{SCCs}$and $S_{ICCs}$are the respective rates of proliferation and degradation for the CCs, SCCs and ICCs, as specified subsequently in Equations (S9-S11).

**Components of the tumor microenvironment**

As outlined in the current mathematical framework, we incorporate the interactions that occur among cancer cells, immune system cells, and tumor-associated macrophages, which will be elaborated upon subsequently.

**Cancer Cells**

The dynamics of the populations of non-stem-like cancer cells (CCs), stem-like cancer cells (SCCs), and induced cancer cells (ICCs) are delineated by Equations (S5-S7). SCCs exhibit resistance to pharmacotherapeutic agents, hypoxic conditions, and the immune response, whereas ICCs, upon the administration of nano-immunotherapy, adopt a more stem-like phenotype.

| $\frac{\partial T_{CCs}}{\partial t}=\nabla\cdot(\mathrm{D}_{CCs}\nabla T_{CCs})+GS_{f}T_{CCs}-cNT_{CCs}-D+p_{CT}T_{SCCs}+p_{IT}T_{ICCs}-\left(p_{TC}+p_{TI}\right)T_{CCs}-\lambda_{M1}M_{1}T_{CCs}$ | (S5) |
| --- | --- |
| $\frac{\partial T_{SCCs}}{\partial t}=\nabla\cdot(D_{SCCs}\nabla T_{SCCs})+\alpha_{SCCs}GS_{f_{SCCs}}T_{SCCs}-c_{SCCs}NC_{SCCs}-D_{SCCs}+p_{TC}T_{CCs}+p_{IC}T_{ICCs}-\left(p_{CT}+p_{CI}\right)T_{SCCs}$ | (S6) |
| $\frac{\partial T_{ICCs}}{\partial t}=\nabla\cdot(\mathrm{D}_{ICCs}\nabla T_{ICCs})+\alpha_{ICCs}GS_{f_{ICCs}}T_{ICCs}-c_{ICCs}NT_{ICCs}-D_{ICCs}+p_{TI}T_{CCs}+p_{CI}T_{SCCs}-\left(p_{IT}+p_{IC}\right)T_{ICCs}$ | (S7) |

In this context, $N$ represents the Natural Killer (NK) cells, $M_{1}$corresponds to the $M_{1}$ tumor-associated macrophages (TAMs), while$\mathrm{D}_{CCs}$,$D_{SCCs}$, and $\mathrm{D}_{ICCs}$ denote the diffusion coefficients of non-stem-like, stem-like, and induced cancer cells, respectively. To incorporate the influence of drug delivery on growth, the surviving fractions of cancer cells, denoted as $S_{f}$, $S_{f_{SCCs}}$, and $S_{f_{ICCs}}$, are integrated into Equations (S5), (S6), and (S7), respectively. The variables$c$, $c_{SCCs}$,$c_{ICCs}$and $D$, $D_{SCCs}$,$D_{ICCs}$ quantify the respective proportions of tumor cells that are eliminated by NK and CD8^+^ T-cells. Especially, for the parameters $c_{SCCs}$, $D_{SCCs}$, $c_{ICCs}$ and $D_{ICCs}$, which characterize the cytotoxic potential of immune cells against SCCs and ICCs, we argue that these cells display heightened resistance during interactions with immune entities. Based on empirical evidence [14], the cytotoxic efficacy of CD8^+^ T-cells against stem-like cancer cells (SCCs) is considered to be seven times lower than that against CCs.  Therefore, the parameters that govern the elimination of SCCs by immune cells are assumed to be the same as for CCs but multiplied by a factor of 0.14. Furthermore, three days later upon sonopermeation treatment, viability of SCCs decreased twice [15].

G characterizes the proliferation of CCs, SCCs, and ICCs as a function of oxygen availability.  Regarding the coefficients of the proliferation rates for SCCs and ICCs, namely $\alpha_{SCCs}$ and $\alpha_{ICCs}$, we claim that under normal oxygen conditions, these values are equivalent to one, thereby ensuring that all cancer cell types exhibit proliferation rates consistent with those of CCs. Conversely, under hypoxic conditions, the proliferation rates of cancer cells exhibiting a stem-like phenotype are observed to increase. Consequently, we hypothesize that their proliferation rates are inversely proportional to the oxygen concentration, such that as the oxygen concentration approaches zero, the proliferation rates double compared to those observed under normal oxygen levels [16]. The transition rates of cancer cells from type i to type j are denoted by $p_{ij}$ [17].  Moreover, the parameter $\lambda_{M1}$ signifies the tumoricidal activity of M1-like TAMs against cancer cells [18].

The relationship between tumor cells proliferation and the local oxygen concentration, $G$, is asserted to follow Michaelis-Menten kinetics, taking the form presented in Equation (S8) [19, 20]:

| $G=\frac{k_{1} c_{ox}}{k_{2}+c_{ox}}$ | (S8) |
| --- | --- |

where $k_{1}$and $k_{2}$ are parameters indicative of the growth rate, and $c_{ox}$represents the concentration of oxygen.

The mechanism underlying the creation and degradation of the solid phases, $S_{CCs}$, $S_{SCCs}$ and $S_{ICCs}$ is delineated as follows Equations (S9-S11):

| $S_{CCs}=GS_{f}T_{CCs}-cNT_{CCs}-D+p_{CT}T_{SCCs}+p_{IT}T_{ICCs}-\left(p_{TC}+p_{TI}\right)T_{CCs}-\lambda_{M1}M_{1}T_{CCs}$ | (S9) |
| --- | --- |
| $S_{SCCs}=\alpha_{SCCs}GS_{f_{SCCs}}T_{SCCs}-c_{SCCs}NC_{SCCs}-D_{SCCs}+p_{TC}T_{CCs}+p_{IC}T_{ICCs}-\left(p_{CT}+p_{CI}\right)T_{SCCs}$ | (S10) |
| $S_{ICCs}=\alpha_{ICCs}GS_{f_{ICCs}}T_{ICCs}-c_{ICCs}NT_{ICCs}-D_{ICCs}+p_{TI}T_{CCs}+p_{CI}T_{SCCs}-\left(p_{IT}+p_{IC}\right)T_{ICCs}$ | (S11) |

**Immune Cells**

For the immune system, this model considers four principal categories of immune cells: natural killer (NK) cells, CD8^+^ T-cells, CD4^+^ T-cells and regulatory T-cell (Treg) subset. Utilizing relevant research work [21-23], the system of equations incorporates the recruitment rates of these immune cells, their inactivation by tumor cells, the suppressive function of Tregs and M2 tumor-associated macrophages (TAMs), as well as their mortality rate and interactions with cancer cells. The following Equations (S12-S15) illustrate the interactions that taking place among the cellular elements of the immune system:

| $\frac{\partial N}{\partial t}=\sigma_{nk}-f_{NK}N+\frac{g_{NK}T_{CCs}^{2}}{h+T_{CCs}^{2}}N-p_{im}NT_{CCs}-\lambda_{reg}T_{reg}N-\lambda_{M2}M_{2}N$ | (S12) |
| --- | --- |
| $\frac{\partial L}{\partial t}=\sigma_{T8}-m_{T8}L+\frac{j_{T8}D^{2}}{k_{im}+D^{2}}L-qLT_{CCs}+(r_{N}N+r_{Cd4}C_{d4})T_{CCs}-\lambda_{reg}T_{reg}L-\lambda_{M2}M_{2}L$ | (S13) |
| $\frac{\partial C_{d4}}{\partial t}=s_{CD4}+re_{Cd4}C_{d4}\left(1-\frac{C_{d4}}{C_{d4,\max}}\right)-\mu_{C_{d4}}C_{d4}$ | (S14) |
| $\frac{\partial T_{reg}}{\partial t}=g_{reg}T_{reg}-m_{reg}T_{reg}$ | (S15) |

where $N$ is the population of NK cells, $L$ of CD8^+^ T-cells, $C_{d4}$ denotes the population of CD4^+^ T-cells and $T_{reg}$ represents the population of regulatory T-cells. Additionally, $f_{NK}$, $m_{T8}$, and $m_{reg}$ signify the mortality rates of NK cells, CD8^+^ T-cells, and Treg cells, respectively, while $g_{NK}$, $j_{T8}$, and $g_{reg}$ denote the recruitment rates of immune cells, and $p_{im}$ and $q$ represent the inactivation rates of immune cells by cancer cells (CCs). The constants $\sigma_{nk}$ and $\sigma_{T8}$ denote the stable sources of NK and CD8^+^ T-cells, respectively, $r_{N}$ indicates the rate at which tumor-specific CD8^+^ T-cells are stimulated for production due to tumor cells being eliminated by NK cells, and $\lambda_{reg}$ serves as the inhibition term affecting NK cells and CD8^+^ T-cells as influenced by Treg cells. Under conditions of hypoxia, the minimum activity levels for NK and CD8^+^ T-cells, were employed, which subsequently increased linearly to the maximum observed levels under normoxic conditions [22]. The values of $f_{NK}$and $m_{T8}$were adjusted to reflect variations in oxygen levels. In accordance with experimental data [24], a 40-fold reduction in oxygen concentration (from 20% to 0.5%) resulted in a twofold increase in the apoptotic rate of immune cells. Furthermore, $s_{CD4}$ serves as the source of CD4^+^ T-cells, $\mu_{C_{d4}}$ represents the natural mortality rate of CD4^+^ T-cells, $re_{Cd4}$ indicates the growth rate of CD4^+^ T-cells, and $C_{d4,\max}$signifies the maximum population of CD4^+^ T-cells [25, 26]. The stimulation rate of CD8^+^ T-cells by CD4^+^ T-cells, as previously noted [27-29], is represented by $r_{Cd4}$. The source term for CD4^+^ T-cells, $s_{CD4}$, is reliant on the concentration of oxygen, as previous studies have indicated an eightfold decrease under hypoxic conditions [30]. Upon sonopermeation, source terms of CD8^+^ and CD4^+^ T-cells increase 1.5 times and 1.2 times [31], respectively. Moreover, a reduction in M2 TAMs led to an increase in the populations of CD8^+^ T-cells and NK cells, while CD4^+^ T-cells remained unaffected, according to experimental findings [32], and these dynamics are articulated by the parameter $\lambda_{M2}$. The equations that describe the cell populations are normalized by transforming them dimensionless through the division of the cell count per finite element node by the initial quantity of cancer cells, T0=5×10² cells. The initial cancer cell population was established as follows: 98% CCs, 1% SCCs, and 1% ICCs [33].

Additionally, the variable $D$ represents the fractional cell kill of tumor cells by CD8^+^ T-cells and is defined by Equation (S16) [22, 34]:

| $D=d_{im}\frac{\left(\frac{L}{T_{CCs}}\right)^{\lambda_{im}}}{s+\left(\frac{L}{T_{CCs}}\right)^{\lambda_{im}}}T_{CCs}$, | (S16) |
| --- | --- |

In this expression, $d_{im}$signifies the saturation threshold of fractional tumor cell eradication by CD8^+^ T-cells, $s$reflects the steepness coefficient associated with the competition between the tumor and CD8^+^ T-cells, and $\lambda_{im}$ denotes the exponent corresponding to fractional tumor cell kill by CD8^+^ T-cells.

**Tumor Associated Macrophages (TAMs)**

In this mathematical model, two distinct classifications of TAMs are recognized, specifically M1 and M2:

| $\begin{matrix} \frac{\partial M_{1}}{\partial t}=g_{m1}M_{1}-m_{m1}M_{1} \\ \frac{\partial M_{2}}{\partial t}=g_{m2}M_{2}-m_{m2}M_{2}+r_{Cvegf,M2}C_{vegf}M_{2} \end{matrix}$ | (S17) |
| --- | --- |

The parameters $g_{m1}$ and $g_{m2}$ signify the production rates for M1and M2 TAMs, which are influenced by oxygen concentrations, as indicated by prior investigations [32, 35, 36] showing that a decline in hypoxia leads to a reorientation in TAM polarization from the M2-like to the M1-like phenotype. Previous investigations have established a correlation between TAMs and the expression of VEGF [32, 37, 38]. In particular, the overexpression of VEGF-A has been linked to an increased presence of M2-like TAMs ($r_{Cvegf,M2}$).

**Implementation of Biphasic Theory for Understanding the Mechanical Behavior of the Tumor**

The conservation principles of both the solid and fluid phases within the tumor system are expressed through the subsequent mass balance equations: [8, 11]

| $\frac{\partial\Phi^{c}}{\partial t}+\nabla\cdot(v^{s}\Phi^{c})=\frac{T_{CCs}}{T_{tot}}S_{CCs}+\frac{T_{SCCs}}{T_{tot}}S_{SCCs}+\frac{T_{ICCs}}{T_{tot}}S_{ICCs}$ | (S18) |
| --- | --- |
| $\frac{\partial\Phi^{f}}{\partial t}+\nabla\cdot(v^{f}\Phi^{f})=Q$ | (S19) |

In this context, $\Phi^{c}$ and $\Phi^{f}$ represent the volume fractions of the solid and fluid phases, respectively, while $v^{s}$ and $v^{f}$ denote their associated velocities.

The variable $Q$ in Equation (S20) signifies the fluid flux entering the tumor from blood vessels as well as from the adjacent normal tissue, subtracted by the fluid flux exiting via lymphatic vessels, and is expressed as [9]:

| $Q=L_{p}S_{v}(p_{v}-p_{i})-L_{pl}S_{vl}(p_{i}-p_{l})$ | (S20) |
| --- | --- |

where $L_{p}$, $S_{v}$, and $p_{v}$ are indicative of the hydraulic conductivity, vascular density, and vascular pressure, respectively; $L_{pl}$, $S_{vl}$, and $p_{l}$ are the analogous parameters for lymphatic vessels; and $p_{i}$corresponds to the interstitial fluid pressure. In this particular model configuration and taking into account the principle of mass conservation within the tissue, the total of the solid and fluid volume fractions is established to consistently amount to one, in accordance with Equation (S21).

| $\Phi^{f}=1-\Phi^{c}$ | (S21) |
| --- | --- |

Moreover, summing Equations (S18) and (S19), the mass balance can be expressed as follows:

| $\nabla\cdot\left(\Phi^{c}v^{s}+\Phi^{f}v^{f}\right)=Q+\frac{T_{CCs}}{T_{tot}}S_{CCs}+\frac{T_{SCCs}}{T_{tot}}S_{SCCs}+\frac{T_{ICCs}}{T_{tot}}S_{ICCs}$ | (S22) |
| --- | --- |

where the fluid velocity $\mathbf{v}^{f}$ is defined according to Darcy’s law [39]

| $\Phi^{f}(v^{f}-v^{s})=-k_{th}\nabla p_{i}Þv^{f}=\frac{-k_{th}\nabla p_{i}}{\Phi^{f}}+v^{s}$ | (S23) |
| --- | --- |

with $k_{th}$denoting the hydraulic conductivity of the interstitial medium [40].

In accordance with the biphasic theory applicable to soft tissues [41], the overall stress tensor $\sigma_{tot}$is constituted by the fluid phase stress tensor $\sigma^{f}=-p_{i}I$ and the solid phase stress tensor $\sigma^{s}$. Consequently, the stress balance can be expressed as:

| $\nabla\cdot\sigma_{tot}=0 \Rightarrow\nabla\cdot(\sigma^{s}-p_{i}I)=0$ | (S24) |
| --- | --- |

where the Cauchy stress tensor associated with the solid phase $\sigma^{s}$ is delineated by: [42]

| $\sigma^{s}=J_{e}^{-1}F_{e}\frac{\partial W}{\partial F_{e}^{T}}$, | (S25) |
| --- | --- |

The mechanical properties of the tumor have been defined as incompressible and neo-Hookean, with the strain energy density represented by: [43-46]

| $W=\frac{1}{2}\mu\left(-3+II_{1}\right)-p\left(J_{e}-1_{1}\right)-\left(\frac{p^{2}}{2k}\right)$ | (S26) |
| --- | --- |

where $\mu$ and $k$ denote the shear and bulk modulus of the material, respectively; $J_{e}$ signifies the determinant of the elastic deformation gradient tensor $F_{e}$; $II_{1}=I_{1}J_{e}^{-2/3}$ with $I_{1}=trC_{e}$being the first invariant of the elastic Cauchy-Green deformation tensor $C_{e}=F_{e}^{T}F_{e}$, and $p$is a penalty variable introduced for materials that exhibit near incompressibility that regularizes the constraint involved in the second term [47]. Values of shear modulus are based on experimental findings from our previous studies [48, 49]. The adjacent normal tissue is presumed to be compressible and neo-Hookean, characterized by a Poisson ratio of 0.2 and tumor tissue with a Poisson ratio of 0.4.

**Functional Vascular Density**

To assess the functional vascular density, it is claimed that is impacted by the decrease in the diameter of blood vessels ($\frac{d}{d_{0}}$) linked to the increase of solid stress [2]. Furthermore, the functional vascular density is dependent on the permeability of the tumor vascular wall [5], as hyper-permeable vessels diminish both perfusion and overall functionality of the vessels.

The functional vascular density can be expressed as:

| $S_{v}=\frac{d}{d_{0}}S_{v}^{0}\rho_{v}^{EC}$, | (S27) |
| --- | --- |

where $S_{v}^{0}$ will be determined by the dimensions of the pores in the vascular wall (i.e., its permeability) and $\rho_{v}^{EC}$ is related to the density of endothelial cells.

**Transport of Oxygen**

The variation in oxygen levels within the tumor tissue is considered to be based on both its transport mechanisms, namely convection and diffusion, alongside the rate of oxygen utilized by the cells, as well as the influx of oxygen from the surrounding blood vessels [7, 8], specifically described by the equation:

| $\frac{\partial c_{ox}}{\partial t}+\nabla\cdot\left(c_{ox}\mathbf{v}^{f}\right)=D_{ox}\nabla^{2}c_{ox}-\frac{A_{ox}c_{ox}}{c_{ox}+k_{ox}}T_{tot}+P_{er}S_{V}\left(C_{iox}-c_{ox}\right)$, | (S28) |
| --- | --- |

where $c_{ox}$ represents the oxygen concentration, $D_{ox}$ denotes the diffusion coefficient for oxygen in the interstitial region, $A_{ox}$ and $k_{ox}$ are parameters  associated with oxygen uptake, $P_{er}$ indicates the vascular permeability of oxygen, which governs the diffusion across the walls of tumor vessels, and $C_{iox}$ is signifies the oxygen concentration present in the vascular system. The transport of oxygen across the vascular barrier is primarily governed by diffusion, as the contribution of convection is minimal in comparison to diffusion [50]. In view of the uniform reduction of interstitial fluid pressure observed in tumors, the pressure differentials both within the tissue and across the tumor vessel wall are minimal [51], leading to an expectation of low Péclet numbers.

**Components of Tumor Vasculature**

In accordance with our mathematical model, we integrate the constituents of tumor vasculature, which include the endothelial cells, the vascular endothelial growth factor (VEGF), as well as Angiopoietin 1 and Angiopoietin 2.

T**ransport Equation of Endothelial Cells**

The flux of endothelial cells is represented by the Equation (S29): [52]

|  | (S29) |
| --- | --- |

The proliferation of endothelial cells is influenced by the concentration of VEGF, as well as the density of endothelial cells. is the dimensionless density of endothelial cells. and $C_{vegf}^{0}$ are dimensionless and refer to VEGF concentrations. The diffusion coefficient of endothelial cells is contingent upon Ang1 and Ang2, described by $D_{EC}(\alpha1,\alpha2)=D_{EC}(1+s1\alpha1)^{-\alpha}(1+s2\alpha2)^{b}$, where $\alpha$ and $b$are set to unity [53], $x_{n}$denotes a chemotactic factor. The dimensionless concentration of endothelial cells is derived by normalizing with the reference concentration, . The constants $\lambda_{2}$ and $\lambda_{4}$ are positive parameters. According to previous study apoptosis of endothelial cells increases 1.3 times 24 hours upon treatment with sonopermeation [54].

T**ransport Equation of VEGF**

The concentration of Vascular Endothelial Growth Factor (VEGF) is influenced by various factors including diffusion, synthesis from cancer cells in hypoxic conditions, and interaction with endothelial cell receptors [52]. The concentration of VEGF is represented by the following Equation (S30):

|  | (S30) |
| --- | --- |

where denotes the dimensionless VEGF concentration achieved by normalizing with a reference value and represents the dimensionless oxygen concentration normalized as: .

It is assumed that VEGF is exclusively synthesized by tumor cells, with its production being intensified in response to hypoxic conditions, as characterized by the oxygen tension parameter $G{}_{\alpha}$ [52].

|  | (S31) |
| --- | --- |

VEGF becomes unavailable through its binding to receptors on endothelial cells, and it is also capable of diffusing within the tumor characterized by a diffusion coefficient $D_{VEGF}$. $\lambda_{10}$, $\lambda_{11}$and $\lambda_{13}$ are defined as positive constants. Furthermore, the removal of CD4^+^ T cells led to a notable upregulation of VEGF () without marked alterations in Ang1-Ang2 levels [28].

T**ransport Equation of Angiopoietin 1(Ang1) and Angiopoietin 2 (Ang2)**

The synthesis of the Angiopoietin 1 (Ang1) and Angiopoietin 2 (Ang2) is augmented under hypoxic conditions, which correlate with levels of vascular endothelial growth factor (VEGF) [53]. Both angiopoietin 1 (Ang1, $\alpha_{1}$) and angiopoietin 2 (Ang2, $\alpha_{2}$) exhibit up-regulation in response to hypoxia and are secreted by endothelial cells.

|  | (S32) |
| --- | --- |
| $\frac{\partial\hat{\alpha}_{2}}{\partial t}=\frac{b_{2}}{\alpha_{2}^{0}}G_{\alpha}(\hat{c}_{ox})\hat{e}e_{0}-\mu_{2}\hat{\alpha}_{2}$ | (S33) |

In this context, $b_{1}$, $b_{2}$, $\mu_{1}$, and $\mu_{2}$ are defined as positive constants. The dimensionless forms of Ang1 and Ang2 are derived through normalization with respect to a reference concentration, $\hat{\alpha}_{1}=\frac{\alpha_{1}}{\alpha_{1}^{0}}$ and $\hat{\alpha}_{2}=\frac{\alpha_{2}}{\alpha_{2}^{0}}$. The term for oxygen tension, denoted as $G_{\alpha}$, corresponds to the parameters utilized for VEGF. To simplify the equations, we omit considerations of the diffusion processes associated with Ang1 and Ang2, as well as their interactions with specific Tie receptors [55, 56].

**Transport of Drugs**

**Calculation of hydraulic conductivity, vascular permeability, reflection coefficient and surviving fractions of tumor cells**

The hydraulic conductivity was determined from the specified relation [57]:

| $L_{p}=\frac{\gamma r_{0}^{2}}{8\eta L_{vw}}$ | (S34) |
| --- | --- |

where $\gamma$ signifies the fraction of vessel wall surface area that is occupied by pores, $r_{0}$represents the pore radius, $\eta$ refers to the viscosity of water at 310K, and $L_{vw}$ indicates the thickness of the vessel wall.

The vascular permeability $P_{er}$ alongside the reflection coefficient $\sigma_{f}$ were ascertained from the following equations:

| $P_{er}=\frac{\gamma HD_{0}}{L_{vw}}$ | (S35) |
| --- | --- |

| $\sigma_{f}=1-W$ | (S36) |
| --- | --- |

respectively, where$H$ and $W$ account for hydrodynamic as well as electrostatic interactions, and $D_{0}$ is recognized as the diffusion coefficient of a particle in an free solution at 310K, as delineated by the Stokes-Einstein relationship:

| $D_{0}=\frac{K_{b}T_{emp}}{6\pi\eta r_{s}}$ | (S37) |
| --- | --- |

where $K_{b}$ represents the Boltzmann constant, $T_{emp}$ denotes temperature, and $r_{s}$ refers to the radius of the diffusing particle. By disregarding electrostatic interactions, $H$and $W$are simplified to [57]:

| $H=\frac{6\pi F}{K_{t}}$ | (S38) |
| --- | --- |

| $W=\frac{F(2-F)K_{s}}{2K_{t}}$ | (S39) |
| --- | --- |

where F is defined as the partition coefficient [57]:

| $F=1-\lambda^{2}$ | (S40) |
| --- | --- |

and $\lambda$ constitutes the ratio of the drug size to the vessel wall pore size. The coefficients $K_{s}$ and $K_{t}$ as presented in Equations (S38) and (S39) are determined by:

| $\left(\begin{matrix} K_{t} \\ K_{s} \end{matrix}\right)=\frac{9}{4}\pi^{2}\sqrt{2}\left(1-\lambda\right)^{-5/2}\left[1+\sum_{n=1}^{2} \left(\begin{matrix} \alpha_{n} \\ b_{n} \end{matrix}\right)\left(1-\lambda\right)^{n}\right]+\sum_{n=0}^{4} \left(\begin{matrix} \alpha_{n+3} \\ b_{n+3} \end{matrix}\right)\lambda^{n}$ | (S41) |
| --- | --- |

In relation to oxygen, $\gamma$ and $H$ in Equation (S35) were established as equal to unity, thereby presuming that oxygen can diffuse through any point of the vessel wall without being impeded by hydrodynamic interactions; $D_{0}$ for oxygen was assumed to be equivalent to the diffusion coefficient within the tissue, specifically:

| $P_{erox}=\frac{D_{0}}{L_{vw}}$ | (S42) |
| --- | --- |

To clarify the impact of drug delivery on tumor growth, the parameter representing the surviving fraction of cells, denoted as $S_{f}$, is incorporated into Equation (S5), such that in the scenario devoid of pharmacological agents, $S_{f}$ is equivalent to unity. The fraction of surviving cells  in relation to the concentration of the drug has been previously assessed through empirical methods for doxorubicin [58], and the resultant data were subsequently fitted to an exponential model as a function of the internalized chemotherapeutic concentration, $c_{\mathrm{int}}$, namely,

| $S_{urf}=\exp(-\omega c_{\mathrm{int}})$ | (S43) |
| --- | --- |

wherein represents a fitting parameter for doxorubicin [59]. In accordance with this mathematical formulation, should we disregard immune cells and suggest that CCs, SCCs, and ICCs are eliminated by chemotherapy, the terms $S_{CCs}$, $S_{SCCs}$ and $S_{ICCs}$ in Equations (S9), (S10) and (S11) would converge to zero, subsequently rendering the growth stretch ratio in Equation (S4) a constant value. It is reasonable to expect that the growth stretch ratio would decrease following the cessation of CCs, SCCs, and ICCs, rather than sustaining a stable value. To account for this, Equation (S43) was modified in the form:

| $S_{f}=2*(\exp(\omega c_{\mathrm{int}})-0.5)$ | (S44) |
| --- | --- |

Given that stem-like cancer cells (SCCs) exhibit chemoresistance, the equation is calibrated to align with empirical observations [60] in order to determine the fitting parameter $\omega$ pertinent to this context ($S_{fSCCs}$).

**References**

1. Ambrosi D, Mollica F. On the mechanics of a growing tumor. International Journal of Engineering Science. 2002;40(12):1297-316. doi: Pii S0020-7225(02)00014-9

Doi 10.1016/S0020-7225(02)00014-9. PubMed PMID: WOS:000176960900002.

2. Mpekris F, Angeli S, Pirentis AP, Stylianopoulos T. Stress-mediated progression of solid tumors: effect of mechanical stress on tissue oxygenation, cancer cell proliferation, and drug delivery. Biomech Model Mechanobiol. 2015;14(6):1391-402. doi: 10.1007/s10237-015-0682-0. PubMed PMID: 25968141; PubMed Central PMCID: PMC4568293.

3. Rodriguez EK, Hoger A, McCulloch AD. Stress-dependent finite growth in soft elastic tissues. J Biomech. 1994;27(4):455-67. doi: 0021-9290(94)90021-3 [pii].

4. Skalak R, Zargaryan S, Jain RK, Netti PA, Hoger A. Compatibility and the genesis of residual stress by volumetric growth. J Math Biol. 1996;34(8):889-914.

5. Stylianopoulos T, Jain RK. Combining two strategies to improve perfusion and drug delivery in solid tumors. Proc Natl Acad Sci U S A. 2013;110((46)):18632-7.

6. Mascheroni P, Carfagna M, Grillo A, Boso D, Schrefler BA. An avascular tumor growth model based on porous media mechanics and evolving natural states. Mathematics and Mechanics of Solids. 2018;23(4):686-712.

7. Kim Y, Stolarska MA, Othmer HG. The role of the microenvironment in tumor growth and invasion. Progress in biophysics and molecular biology. 2011;106(2):353-79. doi: 10.1016/j.pbiomolbio.2011.06.006.

8. Roose T, Netti PA, Munn LL, Boucher Y, Jain RK. Solid stress generated by spheroid growth estimated using a linear poroelasticity model. Microvasc Res. 2003;66(3):204-12. doi: S0026286203000578 [pii].

9. Stylianopoulos T, Martin JD, Snuderl M, Mpekris F, Jain SR, Jain RK. Coevolution of solid stress and interstitial fluid pressure in tumors during progression: Implications for vascular collapse. Cancer research. 2013;73(13):3833-41. doi: 10.1158/0008-5472.can-12-4521.

10. Pirentis AP, Polydorou C, Papageorgis P, Voutouri C, Mpekris F, Stylianopoulos T. Remodeling of extracellular matrix due to solid stress accumulation during tumor growth. Connective tissue research. 2015;56(5):345-54.

11. Voutouri C, Stylianopoulos T. Evolution of osmotic pressure in solid tumors. J Biomech. 2014;47(14):3441-7. doi: 10.1016/j.jbiomech.2014.09.019. PubMed PMID: 25287111; PubMed Central PMCID: PMC4256429.

12. MacLaurin J, Chapman J, Jones GW, Roose T. The buckling of capillaries in solid tumours. Proc R Soc A. 2012;468:4123-45.

13. Mpekris F, Voutouri C, Papageorgis P, Stylianopoulos T. Stress alleviation strategy in cancer treatment: Insights from a mathematical model. Z Angew Math Mech. 2018:1-12.

14. Todaro M, D'Asaro M, Caccamo N, Iovino F, Francipane MG, Meraviglia S, et al. Efficient killing of human colon cancer stem cells by gammadelta T lymphocytes. J Immunol. 2009;182(11):7287-96. doi: 10.4049/jimmunol.0804288. PubMed PMID: 19454726.

15. Blanco B, Palma R, Hurtado M, Jiménez G, Griñán-Lisón C, Melchor J, et al. Modeling low-intensity ultrasound mechanotherapy impact on growing cancer stem cells. Mathematics and Computers in Simulation. 2025;228:87-102.

16. Conley SJ, Gheordunescu E, Kakarala P, Newman B, Korkaya H, Heath AN, et al. Antiangiogenic agents increase breast cancer stem cells via the generation of tumor hypoxia. Proc Natl Acad Sci U S A. 2012;109(8):2784-9. doi: 10.1073/pnas.1018866109. PubMed PMID: 22308314; PubMed Central PMCID: PMC3286974.

17. Goldman A, Majumder B, Dhawan A, Ravi S, Goldman D, Kohandel M, et al. Temporally sequenced anticancer drugs overcome adaptive resistance by targeting a vulnerable chemotherapy-induced phenotypic transition. Nat Commun. 2015;6:6139. doi: 10.1038/ncomms7139. PubMed PMID: 25669750; PubMed Central PMCID: PMC4339891.

18. Mahlbacher G, Curtis LT, Lowengrub J, Frieboes HB. Mathematical modeling of tumor-associated macrophage interactions with the cancer microenvironment. J Immunother Cancer. 2018;6(1):10. doi: 10.1186/s40425-017-0313-7. PubMed PMID: 29382395; PubMed Central PMCID: PMC5791333.

19. Casciari JJ, Sotirchos SV, Sutherland RM. Mathematical modelling of microenvironment and growth in EMT6/Ro multicellular tumour spheroids. Cell Prolif. 1992;25(1):1-22. PubMed PMID: 1540680.

20. Casciari JJ, Sotirchos SV, Sutherland RM. Variations in tumor cell growth rates and metabolism with oxygen concentration, glucose concentration, and extracellular pH. J Cell Physiol. 1992;151(2):386-94. doi: 10.1002/jcp.1041510220. PubMed PMID: 1572910.

21. Burroughs NJ, Oliveira BMPM, Pinto AA, Ferreira M. Immune response dynamics. Mathematical and Computer Modelling. 2011;53(7-8):1410-9. doi: 10.1016/j.mcm.2010.02.040. PubMed PMID: WOS:000287479000006.

22. de Pillis LG, Radunskaya AE, Wiseman CL. A validated mathematical model of cell-mediated immune response to tumor growth. Cancer Research. 2005;65(17):7950-8. doi: 10.1158/0008-5472.CAN-05-0564. PubMed PMID: WOS:000231659500056.

23. Fouchet D, Regoes R. A population dynamics analysis of the interaction between adaptive regulatory T cells and antigen presenting cells. PLoS One. 2008;3(5):e2306. doi: 10.1371/journal.pone.0002306. PubMed PMID: 18509463; PubMed Central PMCID: PMC2386153.

24. Barsoum IB, Smallwood CA, Siemens DR, Graham CH. A mechanism of hypoxia-mediated escape from adaptive immunity in cancer cells. Cancer Res. 2014;74(3):665-74. doi: 10.1158/0008-5472.CAN-13-0992. PubMed PMID: 24336068.

25. Perelson AS, Kirschner DE, De Boer R. Dynamics of HIV infection of CD4+ T cells. Math Biosci. 1993;114(1):81-125. PubMed PMID: 8096155.

26. Culshaw RV, Ruan S. A delay-differential equation model of HIV infection of CD4+ T-cells. Mathematical biosciences. 2000;165(1):27-39.

27. De Palma M, Jain RK. CD4(+) T Cell Activation and Vascular Normalization: Two Sides of the Same Coin? Immunity. 2017;46(5):773-5. doi: 10.1016/j.immuni.2017.04.015. PubMed PMID: 28514684.

28. Tian L, Goldstein A, Wang H, Ching Lo H, Sun Kim I, Welte T, et al. Mutual regulation of tumour vessel normalization and immunostimulatory reprogramming. Nature. 2017;544(7649):250-4. doi: 10.1038/nature21724. PubMed PMID: 28371798; PubMed Central PMCID: PMC5788037.

29. de Pillis LG. Mathematical modeling of the regulatory T cell effects on renal cell carcinoma treatment. 2013.

30. Wang Q, Liu C, Zhu F, Liu F, Zhang P, Guo C, et al. Reoxygenation of hypoxia-differentiated dentritic cells induces Th1 and Th17 cell differentiation. Mol Immunol. 2010;47(4):922-31. doi: 10.1016/j.molimm.2009.09.038. PubMed PMID: 19910049; PubMed Central PMCID: PMC2815172.

31. Liu HL, Hsieh HY, Lu LA, Kang CW, Wu MF, Lin CY. Low-pressure pulsed focused ultrasound with microbubbles promotes an anticancer immunological response. J Transl Med. 2012;10:221. doi: 10.1186/1479-5876-10-221. PubMed PMID: 23140567; PubMed Central PMCID: PMC3543346.

32. Rolny C, Mazzone M, Tugues S, Laoui D, Johansson I, Coulon C, et al. HRG inhibits tumor growth and metastasis by inducing macrophage polarization and vessel normalization through downregulation of PlGF. Cancer Cell. 2011;19(1):31-44. doi: 10.1016/j.ccr.2010.11.009. PubMed PMID: 21215706.

33. Hermann PC, Huber SL, Herrler T, Aicher A, Ellwart JW, Guba M, et al. Distinct populations of cancer stem cells determine tumor growth and metastatic activity in human pancreatic cancer. Cell Stem Cell. 2007;1(3):313-23. doi: 10.1016/j.stem.2007.06.002. PubMed PMID: 18371365.

34. Milberg O, Gong C, Jafarnejad M, Bartelink IH, Wang B, Vicini P, et al. A QSP Model for Predicting Clinical Responses to Monotherapy, Combination and Sequential Therapy Following CTLA-4, PD-1, and PD-L1 Checkpoint Blockade. Sci Rep. 2019;9(1):11286. doi: 10.1038/s41598-019-47802-4. PubMed PMID: 31375756; PubMed Central PMCID: PMC6677731.

35. Huang Y, Snuderl M, Jain RK. Polarization of tumor-associated macrophages: a novel strategy for vascular normalization and antitumor immunity. Cancer Cell. 2011;19(1):1-2. doi: 10.1016/j.ccr.2011.01.005. PubMed PMID: 21251607; PubMed Central PMCID: PMC3037265.

36. Huang Y, Stylianopoulos T, Duda DG, Fukumura D, Jain RK. Benefits of vascular normalization are dose and time dependent--letter. Cancer Res. 2013;73(23):7144-6. doi: 10.1158/0008-5472.CAN-13-1989. PubMed PMID: 24265277; PubMed Central PMCID: PMC3876035.

37. Linde N, Lederle W, Depner S, van Rooijen N, Gutschalk CM, Mueller MM. Vascular endothelial growth factor-induced skin carcinogenesis depends on recruitment and alternative activation of macrophages. J Pathol. 2012;227(1):17-28. doi: 10.1002/path.3989. PubMed PMID: 22262122.

38. Stockmann C, Doedens A, Weidemann A, Zhang N, Takeda N, Greenberg JI, et al. Deletion of vascular endothelial growth factor in myeloid cells accelerates tumorigenesis. Nature. 2008;456(7223):814-8. doi: 10.1038/nature07445; 10.1038/nature07445.

39. Byrne H, Preziosi L. Modelling solid tumour growth using the theory of mixtures. Mathematical medicine and biology : a journal of the IMA. 2003;20(4):341-66.

40. Stylianopoulos T, Yeckel A, Derby JJ, Luo XJ, Shephard MS, Sander EA, et al. Permeability calculations in three-dimensional isotropic and oriented fiber networks. Phys Fluids (1994). 2008;20(12):123601. doi: 10.1063/1.3021477.

41. Mow VC, Kuei SC, Lai WM, Armstrong CG. Biphasic creep and stress relaxation of articular cartilage in compression? Theory and experiments. Journal of Biomechanical Engineering. 1980;102(1):73-84.

42. Taber LA. Theoretical study of Beloussov's hyper-restoration hypothesis for mechanical regulation of morphogenesis. Biomech Model Mechanobiol. 2008;7(6):427-41. doi: 10.1007/s10237-007-0106-x.

43. Ciarletta P. Buckling instability in growing tumor spheroids. Phys Rev Lett. 2013;110:158102.

44. Voutouri C, Mpekris F, Papageorgis P, Odysseos AD, Stylianopoulos T. Role of constitutive behavior and tumor-host mechanical interactions in the state of stress and growth of solid tumors. PLoS One. 2014;9(8):e104717. doi: 10.1371/journal.pone.0104717. PubMed PMID: 25111061; PubMed Central PMCID: PMC4128744.

45. Xu G, Bayly PV, Taber LA. Residual stress in the adult mouse brain. Biomech Model Mechanobiol. 2009;8(4):253-62. doi: 10.1007/s10237-008-0131-4.

46. Xu G, Kemp PS, Hwu JA, Beagley AM, Bayly PV, Taber LA. Opening angles and material properties of the early embryonic chick brain. J Biomech Eng. 2010;132(1):011005. doi: 10.1115/1.4000169. PubMed PMID: 20524743; PubMed Central PMCID: PMC2882656.

47. Holzapfel GA, Gasser TC, Ogden RW. A new constitutive framework for arterial wall mechanics and a comparative study of material models. J Elasticity. 2000;61:1-48.

48. Mpekris F, Panagi M, Charalambous A, Voutouri C, Michael C, Papoui A, et al. A synergistic approach for modulating the tumor microenvironment to enhance nano-immunotherapy in sarcomas. Neoplasia. 2024;51:100990. doi: 10.1016/j.neo.2024.100990. PubMed PMID: 38520790; PubMed Central PMCID: PMC10978543.

49. Panagi M, Mpekris F, Voutouri C, Hadjigeorgiou AG, Symeonidou C, Porfyriou E, et al. Stabilizing Tumor-Resident Mast Cells Restores T-Cell Infiltration and Sensitizes Sarcomas to PD-L1 Inhibition. Clinical cancer research : an official journal of the American Association for Cancer Research. 2024;30(11):2582-97. doi: 10.1158/1078-0432.CCR-24-0246. PubMed PMID: 38578281; PubMed Central PMCID: PMC11145177.

50. Popel AS. Theory of oxygen transport to tissue. Crit Rev Biomed Eng. 1989;17(3):257-321. PubMed PMID: 2673661; PubMed Central PMCID: PMC5445261.

51. Chauhan VP, Stylianopoulos T, Boucher Y, Jain RK. Delivery of molecular and nanomedicine to tumors: Transport barriers and strategies. Annual Reviews Chemical and Biomolecular Engineering. 2011;2:281-98.

52. Schugart RC, Friedman A, Zhao R, Sen CK. Wound angiogenesis as a function of tissue oxygen tension: a mathematical model. Proc Natl Acad Sci U S A. 2008;105(7):2628-33. doi: 10.1073/pnas.0711642105. PubMed PMID: 18272493; PubMed Central PMCID: PMC2268187.

53. Plank MJ, Sleeman BD, Jones PF. The role of the angiopoietins in tumour angiogenesis. Growth Factors. 2004;22(1):1-11. PubMed PMID: 15179939.

54. Haugse R, Langer A, Murvold ET, Costea DE, Gjertsen BT, Gilja OH, et al. Low-Intensity Sonoporation-Induced Intracellular Signalling of Pancreatic Cancer Cells, Fibroblasts and Endothelial Cells. Pharmaceutics. 2020;12(11). doi: 10.3390/pharmaceutics12111058. PubMed PMID: 33171947; PubMed Central PMCID: PMC7694645.

55. Billy F, Ribba B, Saut O, Morre-Trouilhet H, Colin T, Bresch D, et al. A pharmacologically based multiscale mathematical model of angiogenesis and its use in investigating the efficacy of a new cancer treatment strategy. J Theor Biol. 2009;260(4):545-62. doi: 10.1016/j.jtbi.2009.06.026. PubMed PMID: 19615383.

56. Gevertz JL, Torquato S. Modeling the effects of vasculature evolution on early brain tumor growth. J Theor Biol. 2006;243(4):517-31. doi: 10.1016/j.jtbi.2006.07.002. PubMed PMID: 16938311.

57. Deen WM. Hindered Transport of Large molecules in Liquid-Filled Pores. AIChE J. 1987;33(9):1409-25.

58. Kerr DJ, Kerr AM, Freshney RI, Kaye SB. Comparative intracellular uptake of adriamycin and 4'-deoxydoxorubicin by non-small cell lung tumor cells in culture and its relationship to cell survival. Biochem Pharmacol. 1986;35(16):2817-23. PubMed PMID: 3741470.

59. Eikenberry S. A tumor cord model for doxorubicin delivery and dose optimization in solid tumors. Theor Biol Med Model. 2009;6:16. doi: 10.1186/1742-4682-6-16. PubMed PMID: 19664243; PubMed Central PMCID: PMC2736154.

60. Liu G, Yuan X, Zeng Z, Tunici P, Ng H, Abdulkadir IR, et al. Analysis of gene expression and chemoresistance of CD133+ cancer stem cells in glioblastoma. Mol Cancer. 2006;5:67. doi: 10.1186/1476-4598-5-67. PubMed PMID: 17140455; PubMed Central PMCID: PMC1697823.
