# Supplementary material for: Optimizing therapeutic outcomes with Mechanotherapy and Ultrasound Sonopermeation in solid tumors
Source: PLoS Comput Biol. 2025 Sep 23;21(9):e1012676. doi: 10.1371/journal.pcbi.1012676 (PMC12483211; doi:10.1371/journal.pcbi.1012676)
Supplement: S1 Table — (DOCX) [file pcbi.1012676.s002.docx]

**Table S1.** Parameter values applied within the model

| **Description** | **Parameter** | **Value [Units]** | **Reference** |
| --- | --- | --- | --- |
| Fractional tumor cell killing by NK cells | $c$ | range : 3.23×10^−7^ -3.23×10^−6^ [cell^−1^∙day^–1^] | [1] |
| Cell diffusion coefficient | $D_{cell}$ | 1.5x10^-11^ [m^2^/s] | [2, 3] |
| Stem-like-cancer cell growth multiplier | $\alpha_{SCCs}$ | range : 1-2 [-] | [4] |
| Induced cancer cell growth multiplier | $\alpha_{ICCs}$ | range : 1-2 [-] | **---** |
| Rate of transition from non-stem-like cancer cells to stem-like cancer cells | $p_{TC}$ | 0.55 [day^–1^] | [5] |
| Rate of transition from stem-like cancer cells to non-stem-like cancer cells | $p_{CT}$ | 1 [day^–1^] | [5] |
| Rate of transition from stem-like cancer cells to induced cancer cells | $p_{CI}$ | 0.58 [day^–1^] | [5] |
| Rate of transition from induced cancer cells to stem-like cancer cells | $p_{IC}$ | 0.96 [day^–1^] | [5] |
| Rate of transition from non-stem-like cancer cells to induced cancer cells | $p_{TI}$ | 0.21 [day^–1^] | [5] |
| Rate of transition from induced cancer cells to non-stem-like cancer cells | $p_{IT}$ | 1 [day^–1^] | [5] |
| Tumoricidal effect of M1 TAMs in cancer cells | $\lambda_{M1}$ | 3 [d^–1^] | [6] |
| Growth rate parameter | $k_{1}$ | 0.56 [day^−1^] | **---** |
| Growth rate parameter | $k_{2}$ | 0.0083 [mol∙m^−3^] | [7] |
| Constant source of NK cells | $\sigma_{nk}$ | 1.3×10^4^ [cell∙day^–1^] | [1] |
| Death rate of NK cells | $f_{NK}$ | range0.0412 - 0.0814 [day^–1^] | [1] |
| Recruitment rate of NK cells | $g_{NK}$ | 0.025 [day^–1^] | [1] |
| Steepness coefficient of NK cell recruitment curve | $h$ | 2.02×10^7^ [cell^2^] | [1] |
| Inactivation rate of NK cells | $p_{im}$ | 1×10^−7^ [cell^−1^∙day^–1^] | [1] |
| Inhibition term of NK cells and CD8^+^ T-cells from Treg cells | $\lambda_{reg}$ | 100 [cell^−1^∙day^–1^] | [8] |
| Death rate of CD8^+^ T-cells | $m_{T8}$ | range : 0.02 - 0.04 [day^-1^] | [1] |
| Death rate of regulatory T-cells | $m_{reg}$ | 0.02 [day^–1^] | [8] |
| Recruitment rate of CD8^+^ T-cells | $j_{T8}$ | 0.0375 [day^–1^] | [1] |
| Recruitment rate of regulatory T-cells | $g_{reg}$ | 0.0375 [day^–1^] | [8] |
| Steepness coefficient of CD8^+^ T-cells recruitment curve | $k_{im}$ | 2.02×10^7^ [cell^2^] | [1] |
| Inactivation rate of CD8^+^ T-cells | $q$ | 3.42×10^−10^ [cell^−1^∙day^–1^] | [1] |
| Stimulation rate of CD8^+^ T-cells | $r$ | 1.1×10^−7^ [cell^−1^∙day^–1^] | [1] |
| Source term of CD4^+^ T- cells | $s_{CD4}$ | 150 [day^–1^]  | [9] |
| Natural death rate of CD4^+^ T-cells | $\mu_{C_{d4}}$ | 0.02 [day^–1^] | [9] |
| Growth rate of CD4^+^ T-cells | $re_{Cd4}$ | 0.03 [day^–1^] | [9] |
| Stimulation rate of CD8^+^ T cells by CD4^+^ T-cells | $r_{Cd4}$ | 1x10^-15^ [cells^-1^.day^-1^] | [1] |
| Source term of CD8^+^ T- cells | $\sigma_{T8}$ | 150 [day^–1^] | **----** |
| Fractional tumor cell killing by CD8^+^ T-cells | $d_{im}$ | range: 1.43 – 7.15 [day^–1^] | [1] |
| Exponent of fractional cell kill by CD8^+^ T-cells | $\lambda_{im}$ | 1.36 [-] | [1] |
| Steepness coefficient of the tumor-CD8^+^ T-cells competition term | $s$ | 2.73 [-] | [1] |
| Death rate of regulatory M1 TAMs | $m_{m1}$ | 0.02 [day^–1^] | **----** |
| Death rate of regulatory M2 TAMs | $m_{m2}$ | 0.02 [day^–1^] | **----** |
| Initial oxygen concentration | $C_{iox}$ | 0.2 [mol∙m^−3^] | [10] |
| Oxygen diffusion coefficient | D_ox_ | 1.55×10^−4^ [m^2^∙day^−1^] | [11] |
| Oxygen uptake | $A_{ox}$ | 2200 [mol∙m^−3^∙day^−1^] | [7, 11] |
| Oxygen uptake | $k_{ox}$ | 0.00464 [mol∙m^−3^] | [7, 11] |
| Endothelial cell diffusion coefficient | $D_{EC}$ | 1x10^-15^ [m^2^/s] | [12] |
| Chemotactic endothelial cell | $x_{n}$ | 2x10^-15^ [m^5^/kg-s] | [13] |
| Reference value of endothelial cell | $e_{0}$ | 1x10^-3^ [g/cm^3^] | [13] |
| Positive parameter | $\lambda_{2}$ | 1x10^-5^ [cm^3^/g-s] | [13] |
| Positive parameter | $\lambda_{4}$ | 1x10^-1^ [cm^3^/g-s] | [13] |
| Positive parameter | $s1$ | 1x10^3^ [cm^3^/ g] | [12] |
| Positive parameter | $s2$ | 1x10^3^ [cm^3^/g] | [12] |
| VEGF diffusion coefficient | $D_{VEGF}$ | 3.1x10^-11^ [m^2^/s] | [13] |
| Reference VEGF concentration | $C_{vegf}^{0}$ | 1x10^-3^ [g/cm^3^] | [13] |
| Positive parameter | $\lambda_{10}$ | 6.8x10^-3^ [1/s] | [13] |
| Positive parameter | $\lambda_{11}$ | 4 [cm^3^/g-s] | [13] |
| Positive parameter | $\lambda_{13}$ | 4x10^-5^ [1/s] | [13] |
| Reference $\alpha_{1}$ concentration | $\alpha_{1}^{0}$ | 1x10^-3^ [g/cm^3^] | [12] |
| Reference $\alpha_{2}$ concentration | $\alpha_{2}^{0}$ | 1x10^-3^ [g/cm^3^] | [12] |
| Positive parameter | $b_{1}$ | 2280 [1/h] | [12] |
| Positive parameter | $b_{2}$ | 18240 [1/h] | [12] |
| Positive parameter | $\mu_{1}$ | 456 [1/h] | [12] |
| Positive parameter | $\mu_{2}$ | 456 [1/h] | [12] |
| Hydraulic conductivity | $k_{th}$ | 6.5×10^−11^ [m^2^∙Pa^−1^∙day^−1^] for the host tissue,  6.5×10^−11^ [m^2^∙Pa^−1^∙day^−1^] for the tumor,  6.5×10^−9^ [m^2^∙Pa^−1^∙day^−1^] for the tumor with the effect of ketotifen | [14, 15] |
| Shear modulus | $\mu$ | 21 [kPa] for the host tissue,  60 [kPa] for the tumor,  30 [kPa] for the tumor with the effect of ketotifen | [14, 16-19] |
| Bulk modulus | $k$ | 28 [kPa] for the host tissue,  580 [kPa] for the tumor,  290 [kPa] for the tumor with the effect of ketotifen | [14, 16-19] |
| Internalization rate of the drug by the cells | $k_{\mathrm{int}}$ | 3.7 [day^–1^] | [20, 21] |
| Rate constant for the chemotherapy release | $k_{el}$ | 0.181[day^–1^] | ---- |
| Degradation rate of the chemotherapeutic agent | $k_{\deg}$ | 0.02[1/h] | [22] |
| Degradation rate constant of the free antibody | $k_{\deg_{i}}$ | 0.002[1/h] | adaptation to the model's data |
| Chemotherapy molecules contained in  the nanocarrier | $\alpha$ | 10^4^ | [23] |
| Radius of the diffusing nanoparticle particle | $r_{s}$ | 50x10^-9^ [m] | [24] |
| Radius of the antibody (anti-PD-L1) used in immunotherapy |  | 6.35 x10^-9^ [m] | [25] |
| Drug diffusion coefficient | $D_{f}$ | 8.64×10^−6^ [m^2^∙day^−1^] | [24] |
| Nanoparticle diffusion coefficient | $D_{n}$ | 8.64 × 10^−6^ [m^2^ ∙ day^−1^] for 1[nm] drug;  8.64× 10^−8^ [m^2^ ∙ day^−1^] for 50 [nm] drug | [24] |
| Cancer cell survival constant | $\omega$ | 0.6603 [m^3^/mol] | [26] |
| Stem-like cell survival constant | $\omega_{SCCs}$ | 0.0272 [m^3^/mol] | [26, 27] |
| Induced cancer cell survival constant | $\omega_{ICCs}$ | 0.0272 [m^3^/mol] | ---- |
| Blood circulation decay | $k_{d}$ | 0.417 [day^−1^] | [28] |
| Vessel wall thickness | $L_{vw}$ | 5×10^−6^ [m] | [29] |
| Water viscosity at 310K | $\eta$ | 7×10^−4^ [Pa∙s] | [29] |
| Absolute temperature | $T_{emp}$ | 310 [K] | ---- |
| Fraction of vessel wall surface area occupied by pores | $\gamma$ | 1×10^−5^ [-] | [28] |
| Mechanical Index of transducer | $MI$ | 0.34 | [18] |
| Frequency used for sonopermeation | $fr$ | 2.2 [MHz] | [30] |

 : linear increase from minimum to maximum value depending on oxygen levels

: linear decrease from maximum to minimum value depending on oxygen levels

**References**

1. de Pillis LG, Radunskaya AE, Wiseman CL. A validated mathematical model of cell-mediated immune response to tumor growth. Cancer Research. 2005;65(17):7950-8. doi: 10.1158/0008-5472.CAN-05-0564. PubMed PMID: WOS:000231659500056.

2. Tracqui P. From passive diffusion to active cellular migration in mathematical models of tumour invasion. Acta Biotheor. 1995;43(4):443-64. doi: 10.1007/bf00713564. PubMed PMID: 8919353.

3. Voutouri C, Kirkpatrick ND, Chung E, Mpekris F, Baish JW, Munn LL, et al. Experimental and computational analyses reveal dynamics of tumor vessel cooption and optimal treatment strategies. Proc Natl Acad Sci U S A. 2019;116(7):2662-71. doi: 10.1073/pnas.1818322116. PubMed PMID: 30700544; PubMed Central PMCID: PMC6377457.

4. Burroughs NJ, Oliveira BMPM, Pinto AA, Ferreira M. Immune response dynamics. Mathematical and Computer Modelling. 2011;53(7-8):1410-9. doi: 10.1016/j.mcm.2010.02.040. PubMed PMID: WOS:000287479000006.

5. Goldman A, Majumder B, Dhawan A, Ravi S, Goldman D, Kohandel M, et al. Temporally sequenced anticancer drugs overcome adaptive resistance by targeting a vulnerable chemotherapy-induced phenotypic transition. Nat Commun. 2015;6:6139. doi: 10.1038/ncomms7139. PubMed PMID: 25669750; PubMed Central PMCID: PMC4339891.

6. Mahlbacher G, Curtis LT, Lowengrub J, Frieboes HB. Mathematical modeling of tumor-associated macrophage interactions with the cancer microenvironment. J Immunother Cancer. 2018;6(1):10. doi: 10.1186/s40425-017-0313-7. PubMed PMID: 29382395; PubMed Central PMCID: PMC5791333.

7. Casciari JJ, Sotirchos SV, Sutherland RM. Variations in tumor cell growth rates and metabolism with oxygen concentration, glucose concentration, and extracellular pH. J Cell Physiol. 1992;151(2):386-94. doi: 10.1002/jcp.1041510220. PubMed PMID: 1572910.

8. Fouchet D, Regoes R. A population dynamics analysis of the interaction between adaptive regulatory T cells and antigen presenting cells. PLoS One. 2008;3(5):e2306. doi: 10.1371/journal.pone.0002306. PubMed PMID: 18509463; PubMed Central PMCID: PMC2386153.

9. Perelson AS, Kirschner DE, De Boer R. Dynamics of HIV infection of CD4+ T cells. Math Biosci. 1993;114(1):81-125. PubMed PMID: 8096155.

10. Mpekris F, Baish JW, Stylianopoulos T, Jain RK. Role of vascular normalization in benefit from metronomic chemotherapy. Proc Natl Acad Sci U S A. 2017;114(8):1994-9. doi: 10.1073/pnas.1700340114. PubMed PMID: 28174262; PubMed Central PMCID: PMC5338413.

11. Kim Y, Stolarska MA, Othmer HG. The role of the microenvironment in tumor growth and invasion. Progress in biophysics and molecular biology. 2011;106(2):353-79. doi: 10.1016/j.pbiomolbio.2011.06.006.

12. Plank MJ, Sleeman BD, Jones PF. The role of the angiopoietins in tumour angiogenesis. Growth Factors. 2004;22(1):1-11. PubMed PMID: 15179939.

13. Schugart RC, Friedman A, Zhao R, Sen CK. Wound angiogenesis as a function of tissue oxygen tension: a mathematical model. Proc Natl Acad Sci U S A. 2008;105(7):2628-33. doi: 10.1073/pnas.0711642105. PubMed PMID: 18272493; PubMed Central PMCID: PMC2268187.

14. Netti PA, Berk DA, Swartz MA, Grodzinsky AJ, Jain RK. Role of extracellular matrix assembly in interstitial transport in solid tumors. Cancer Res. 2000;60(9):2497-503.

15. Papageorgis P, Polydorou C, Mpekris F, Voutouri C, Agathokleous E, Kapnissi-Christodoulou CP, et al. Tranilast-induced stress alleviation in solid tumors improves the efficacy of chemo- and nanotherapeutics in a size-independent manner. Sci Rep. 2017;7:46140. doi: 10.1038/srep46140. PubMed PMID: 28393881; PubMed Central PMCID: PMC5385877.

16. Eder M, Raith S, Jalali J, Volf A, Settles M, Machens HG, et al. Comparison of different material models to simulate 3-d breast deformations using finite element analysis. Ann Biomed Eng. 2014;42(4):843-57. doi: 10.1007/s10439-013-0962-8. PubMed PMID: 24346816.

17. Samani A, Zubovits J, Plewes D. Elastic moduli of normal and pathological human breast tissues: an inversion-technique-based investigation of 169 samples. Phys Med Biol. 2007;52(6):1565-76. doi: 10.1088/0031-9155/52/6/002. PubMed PMID: 17327649.

18. Mpekris F, Panagi M, Charalambous A, Voutouri C, Michael C, Papoui A, et al. A synergistic approach for modulating the tumor microenvironment to enhance nano-immunotherapy in sarcomas. Neoplasia. 2024;51:100990. doi: 10.1016/j.neo.2024.100990. PubMed PMID: 38520790; PubMed Central PMCID: PMC10978543.

19. Panagi M, Mpekris F, Voutouri C, Hadjigeorgiou AG, Symeonidou C, Porfyriou E, et al. Stabilizing Tumor-Resident Mast Cells Restores T-Cell Infiltration and Sensitizes Sarcomas to PD-L1 Inhibition. Clinical cancer research : an official journal of the American Association for Cancer Research. 2024;30(11):2582-97. doi: 10.1158/1078-0432.CCR-24-0246. PubMed PMID: 38578281; PubMed Central PMCID: PMC11145177.

20. Mok W, Stylianopoulos T, Boucher Y, Jain RK. Mathematical modeling of herpes simplex virus distribution in solid tumors: implications for cancer gene therapy. Clin Cancer Res. 2009;15(7):2352-60. doi: 1078-0432.CCR-08-2082 [pii] 10.1158/1078-0432.CCR-08-2082.

21. Schmidt MM, Wittrup KD. A modeling analysis of the effects of molecular size and binding affinity on tumor targeting. Molecular cancer therapeutics. 2009;8(10):2861-71. doi: 10.1158/1535-7163.mct-09-0195; 10.1158/1535-7163.mct-09-0195.

22. Wu DC, Ofner CM. Adsorption and degradation of doxorubicin from aqueous solution in polypropylene containers. AAPS PharmSciTech. 2013;14:74-7.

23. Dawidczyk CM, Kim C, Park JH, Russell LM, Lee KH, Pomper MG, et al. State-of-the-art in design rules for drug delivery platforms: lessons learned from FDA-approved nanomedicines. J Control Release. 2014;187:133-44. doi: 10.1016/j.jconrel.2014.05.036. PubMed PMID: 24874289; PubMed Central PMCID: PMC4132889.

24. Pluen A, Boucher Y, Ramanujan S, McKee TD, Gohongi T, di Tomaso E, et al. Role of tumor-host interactions in interstitial diffusion of macromolecules: cranial vs. subcutaneous tumors. Proc Natl Acad Sci U S A. 2001;98(8):4628-33. doi: 10.1073/pnas.081626898 081626898 [pii].

25. Tykodi SS, Brahmer JR, Hwu W-J, Chow LQ, Topalian SL, Hwu P, et al. PD-1/PD-L1 pathway as a target for cancer immunotherapy: Safety and clinical activity of BMS-936559, an anti-PD-L1 antibody, in patients with solid tumors. American Society of Clinical Oncology; 2012.

26. Eikenberry S. A tumor cord model for doxorubicin delivery and dose optimization in solid tumors. Theor Biol Med Model. 2009;6:16. doi: 10.1186/1742-4682-6-16. PubMed PMID: 19664243; PubMed Central PMCID: PMC2736154.

27. Liu G, Yuan X, Zeng Z, Tunici P, Ng H, Abdulkadir IR, et al. Analysis of gene expression and chemoresistance of CD133+ cancer stem cells in glioblastoma. Mol Cancer. 2006;5:67. doi: 10.1186/1476-4598-5-67. PubMed PMID: 17140455; PubMed Central PMCID: PMC1697823.

28. Chauhan VP, Stylianopoulos T, Martin JD, Popovic Z, Chen O, Kamoun WS, et al. Normalization of tumour blood vessels improves the delivery of nanomedicines in a size-dependent manner. Nature Nanotechnology. 2012;7:383-8.

29. Stylianopoulos T, Martin JD, Snuderl M, Mpekris F, Jain SR, Jain RK. Coevolution of solid stress and interstitial fluid pressure in tumors during progression: Implications for vascular collapse. Cancer research. 2013;73(13):3833-41. doi: 10.1158/0008-5472.can-12-4521.

30. Snipstad S, Sulheim E, de Lange Davies C, Moonen C, Storm G, Kiessling F, et al. Sonopermeation to improve drug delivery to tumors: from fundamental understanding to clinical translation. Expert Opin Drug Deliv. 2018;15(12):1249-61. doi: 10.1080/17425247.2018.1547279. PubMed PMID: 30415585.
