# Supplementary material for: Optimizing therapeutic outcomes with Mechanotherapy and Ultrasound Sonopermeation in solid tumors
Source: PLoS Comput Biol. 2025 Sep 23;21(9):e1012676. doi: 10.1371/journal.pcbi.1012676 (PMC12483211; doi:10.1371/journal.pcbi.1012676)
Supplement: S2 Table — (DOCX) [file pcbi.1012676.s003.docx]

**Table S2.** The value of the parameter $k_{1}$, which is employed in the process of fitting the mathematical model to the experimental data for each cancer cell line.

| **Experimental study** | $k_{1}$ |
| --- | --- |
| MCA205-fibrosarcoma tumor cells [1] | 0.56 day^−1^ |
| K7M2-osteosarcoma tumor cells [1] | 0.32 day^−1^ |

**References**

1. Mpekris F, Panagi M, Charalambous A, Voutouri C, Michael C, Papoui A, et al. A synergistic approach for modulating the tumor microenvironment to enhance nano-immunotherapy in sarcomas. Neoplasia. 2024;51:100990. doi: 10.1016/j.neo.2024.100990. PubMed PMID: 38520790; PubMed Central PMCID: PMC10978543.
