# Supplementary material for: Optimizing therapeutic outcomes with Mechanotherapy and Ultrasound Sonopermeation in solid tumors
Source: PLoS Comput Biol. 2025 Sep 23;21(9):e1012676. doi: 10.1371/journal.pcbi.1012676 (PMC12483211; doi:10.1371/journal.pcbi.1012676)
Supplement: S3 Table — (DOCX) [file pcbi.1012676.s004.docx]

**Table S3.** The initial values of the variables used in the mathematical model at time t=0 day.

| **Variable Name** | **Initial Value [Units]** | **Reference** |
| --- | --- | --- |
| Tumor cell populations | | |
| Non-stem-like cancer cells (CCs, dimensionless) | 0.96 [1] | [1] |
| Stem-like-cancer cells (SCCs, dimensionless) | 0.02 [1] | [1] |
| Induced-cancer cells (ICCs, dimensionless) | 0.02 [1] | [1] |
| Immune cell populations | | |
| Natural killer cells (NK, dimensionless) | 0 [1] | [2] |
| CD8^+^ T-cells (dimensionless) | 0 [1] | [3] |
| CD4^+^ T-cells (dimensionless) | 0 [1] | [2] |
| Regulatory T-cells (Treg) (dimensionless) | 0.01 [1] | [2] |
| Tumor Associated Macrophages (TAMs) | | |
| M1 (dimensionless) | 0.01 [-] | [2] |
| M2 (dimensionless) | 0.01 [1] | [2] |
| Tumor vasculature components | | |
| Angiopoietin 1(Ang1, dimensionless) | 0 [1] | [1] |
| Angiopoietin 2 (Ang2, dimensionless) | 0 [1] | [1] |
| Endothelial Cells (ECs, dimensionless) | 0.5 [1] | [1] |
| Vascular Endothelial Growth Factor (VEGF, dimensionless) | 0 [1] | [1] |
| Nanotherapy | | |
| Nanoparticle concentration (c_n_) | 0 [mol/m^3^] | [4] |
| Free chemotherapeutic agent concentration (c_f_) | 0 [mol/m^3^] | [4] |
| Internalized chemotherapeutic agent concentration (c_int_) | 0 [mol/m^3^] | [4] |
| Immunotherapy | | |
| Free immunotherapeutic agent concentration (c_fi_) | 0 [mol/m^3^] | This study |
| Other variables | | |
| Oxygen concentration (c_ox_) | 0.2 [ mol/m^3^] | [4] |
| Interstitial Fluid Pressure (p) | 0 [Pa] | [3] |
| Growth stretch ratio (λ_g_) | 1 [1] | [3] |
| Solid mechanics, displacement vector (**u**) | 0 [m] | [3] |

**References**

1. Mpekris F, Voutouri C, Baish JW, Duda DG, Munn LL, Stylianopoulos T, et al. Combining microenvironment normalization strategies to improve cancer immunotherapy. Proc Natl Acad Sci U S A. 2020;117(7):3728-37. doi: 10.1073/pnas.1919764117. PubMed PMID: 32015113; PubMed Central PMCID: PMC7035612.

2. Mpekris F, Voutouri C, Panagi M, Baish JW, Jain RK, Stylianopoulos T. Normalizing tumor microenvironment with nanomedicine and metronomic therapy to improve immunotherapy. J Control Release. 2022;345:190-9. doi: 10.1016/j.jconrel.2022.03.008. PubMed PMID: 35271911; PubMed Central PMCID: PMC9168447.

3. Hadjigeorgiou AG, Stylianopoulos T. Hybrid model of tumor growth, angiogenesis and immune response yields strategies to improve antiangiogenic therapy. npj Biological Physics and Mechanics. 2024;1(1):4. doi: 10.1038/s44341-024-00002-2.

4. Mpekris F, Baish JW, Stylianopoulos T, Jain RK. Role of vascular normalization in benefit from metronomic chemotherapy. Proc Natl Acad Sci U S A. 2017;114(8):1994-9. doi: 10.1073/pnas.1700340114. PubMed PMID: 28174262; PubMed Central PMCID: PMC5338413.
