# Supplementary figures and images for: Optimizing therapeutic outcomes with Mechanotherapy and Ultrasound Sonopermeation in solid tumors
Source: PLoS Comput Biol. 2025 Sep 23;21(9):e1012676. doi: 10.1371/journal.pcbi.1012676 (PMC12483211; doi:10.1371/journal.pcbi.1012676)

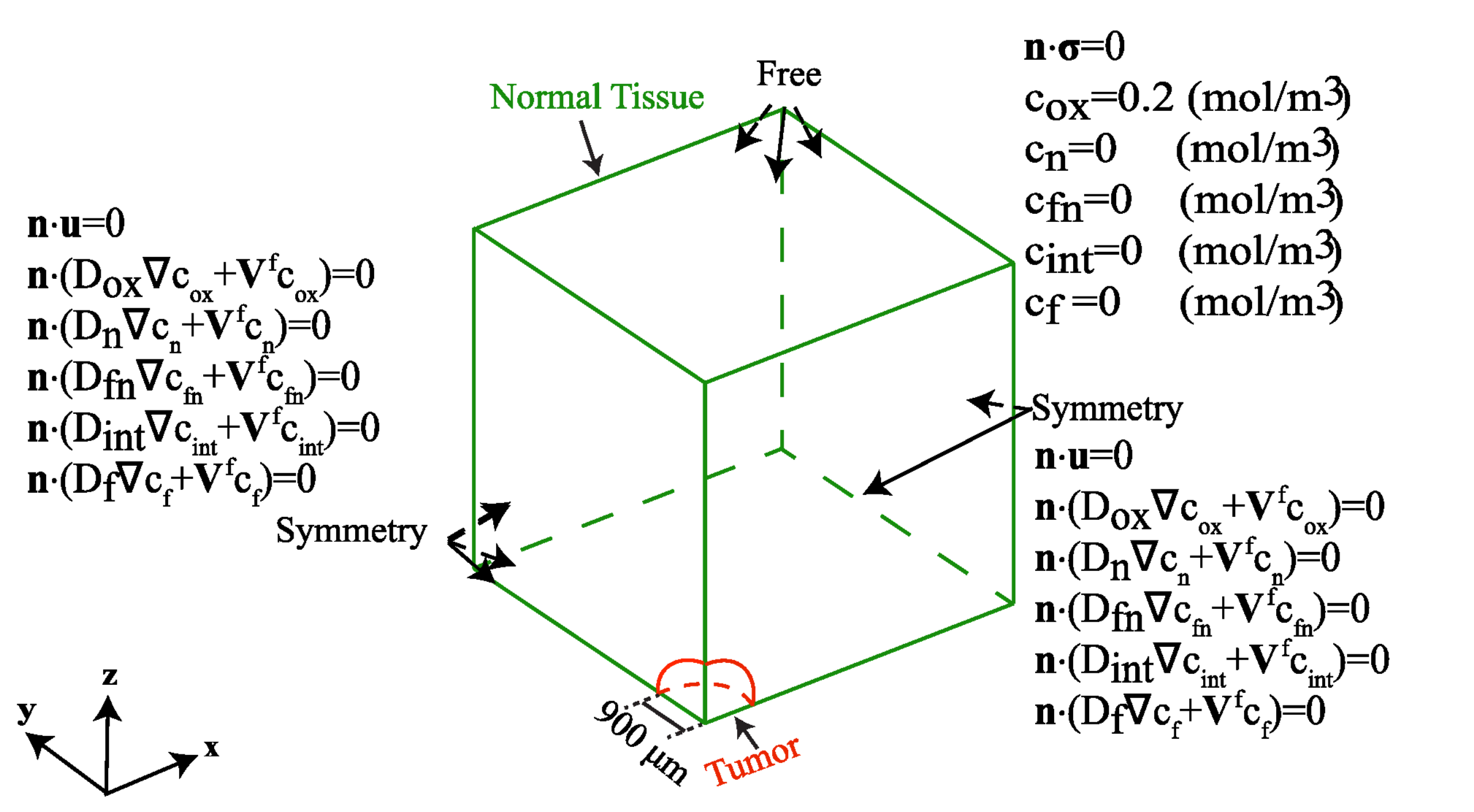

Supplement: S1 Fig — (TIF) [file pcbi.1012676.s005.tif]

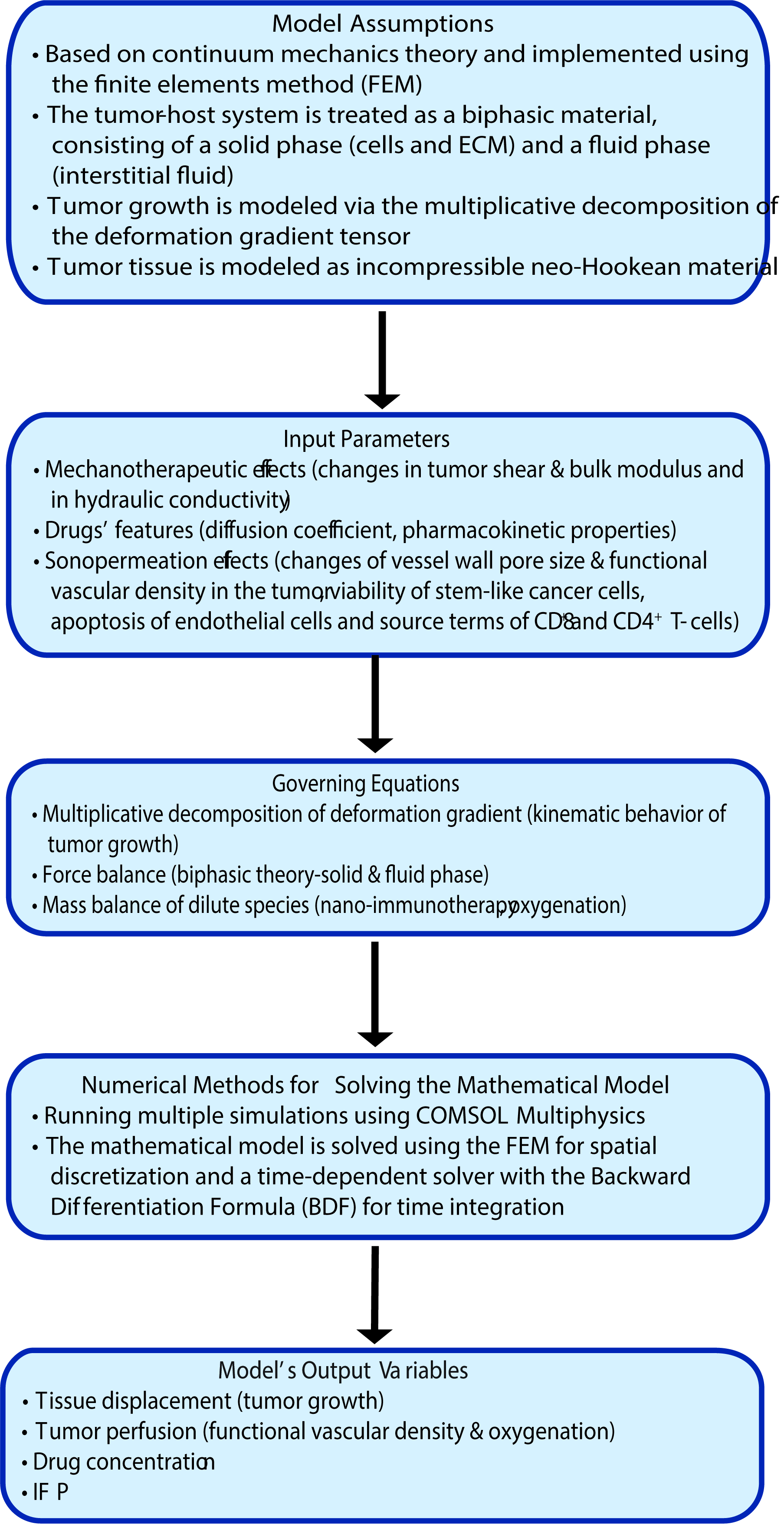

Supplement: S2 Fig — The diagram illustrates the overall computational framework developed to evaluate therapeutic outcomes achieved through mechanotherapy and sonopermeation in solid tumors. It outlines the key assumptions of the model and the main input parameters incorporated into the mathematical formulation. The flowchart also presents the governing equations underlying the mathematical model, along with the numerical methods employed for their solution. Finally, the workflow highlights the resulting output variables that are used to assess therapeutic efficacy. (TIF) [file pcbi.1012676.s006.tif]

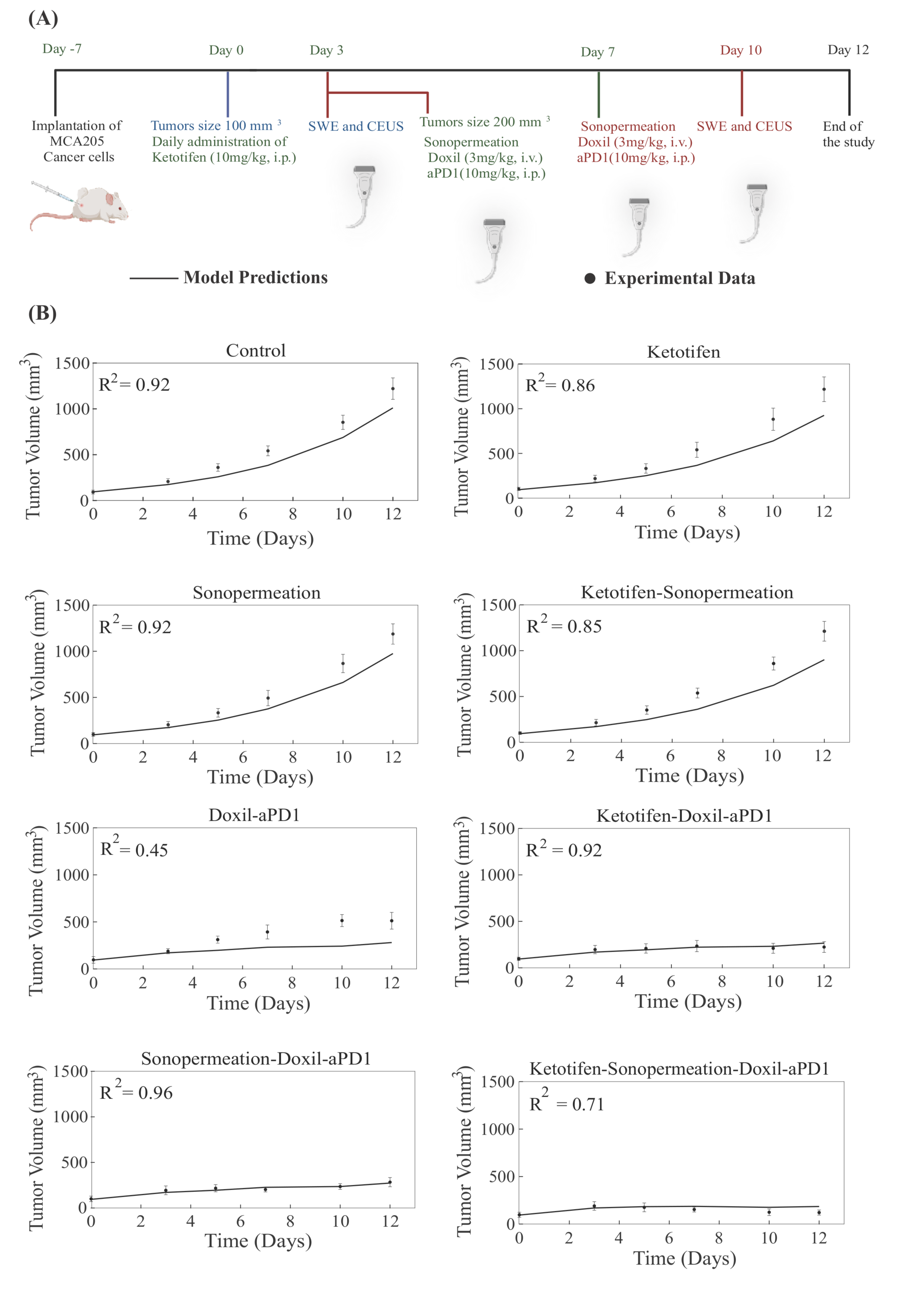

Supplement: S3 Fig — In these simulations, the host tissue is assigned a Poisson’s ratio of ν = 0.49, while the tumor tissue is modeled as incompressible, with a Poisson’s ratio of ν = 0.499999. (A) Experimental treatment protocol followed for MCA205 fibrosarcoma tumors and simulated by the model. Created in BioRender.com. (B) Tumor volume growth rates of murine fibrosarcoma cancer cells (dots) and mathematical model predictions (solid lines) for each treatment group. For each case - control, ketotifen, sonopermeation, ketotifen-sonopermeation, Doxil-aPD1, ketotifen-Doxil-aPD1, sonopermeation-Doxil-aPD1 and ketotifen-sonopermeation-Doxil-aPD1- the R-Squared (R2) value has been calculated and depicts the accuracy of mathematical model validations for tumor growth in comparison with experimental findings. aPD1 denotes for anti-PD1 antibody. We note that increasing the values of the Poisson’s ratio of the tumor and host tissue does not affect qualitatively our results and the model can still provide a good fit to the experimental data. (TIF) [file pcbi.1012676.s007.tif]

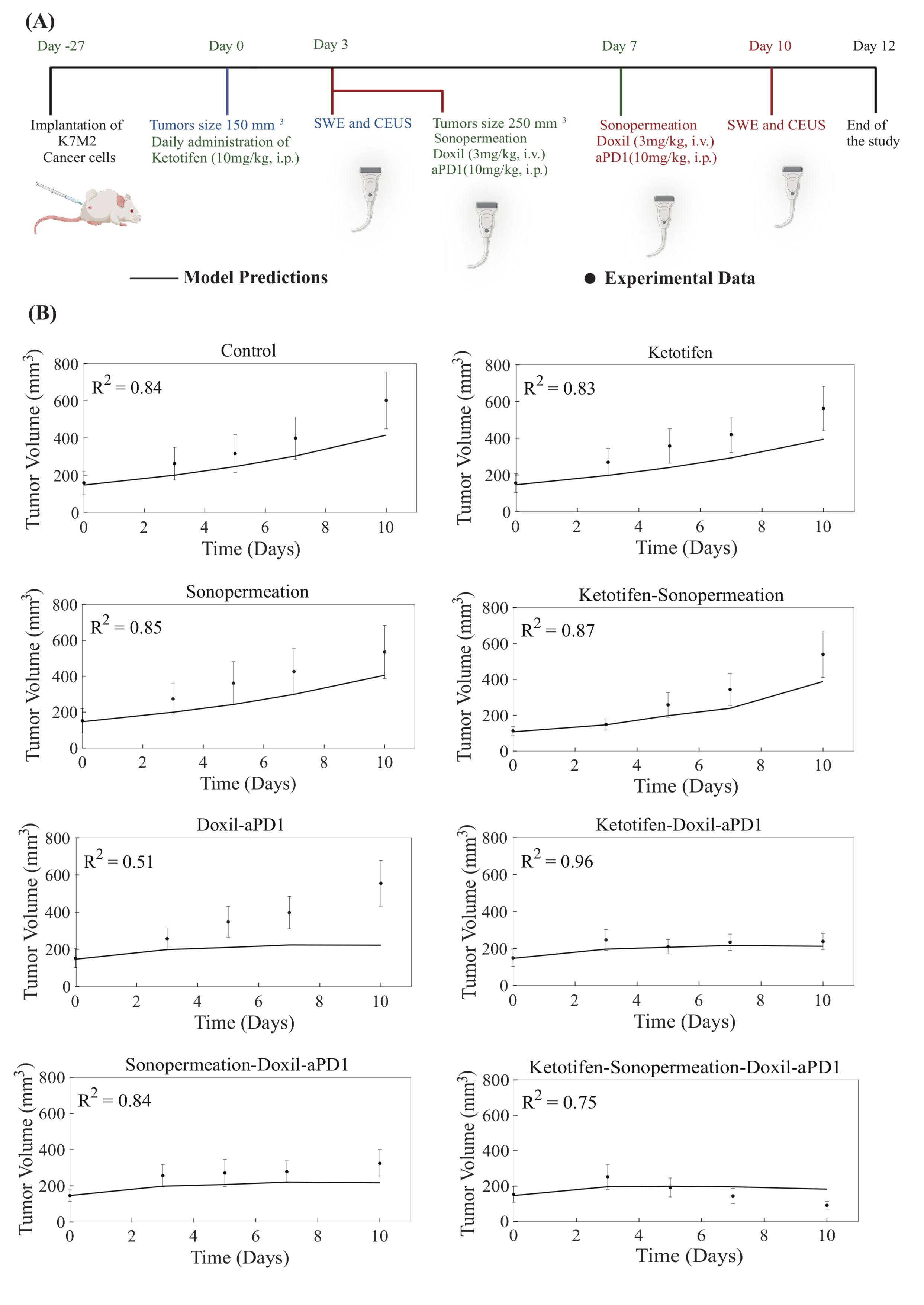

Supplement: S4 Fig — In these simulations, the host tissue is assigned a Poisson’s ratio of ν = 0.49, while the tumor tissue is modeled as incompressible, with a Poisson’s ratio of ν = 0.499999. (A) The experimental treatment protocol implemented for K7M2 osteosarcoma tumors and simulated by the model. Created with BioRender.com. (B) The tumor volume growth of murine osteosarcoma cells (dots) along with the predictions derived from mathematical modeling (solid lines) for each treatment group. For each case - control, ketotifen, sonopermeation, ketotifen-sonopermeation, Doxil-aPD1, ketotifen-Doxil-aPD1, sonopermeation-Doxil-aPD1 and ketotifen-sonopermeation-Doxil-aPD1- the R-Squared (R2) value has been calculated and depicts the accuracy of mathematical model validations for tumor growth in comparison with experimental findings. aPD1 denotes for anti-PD1 antibody. We note that increasing the values of the Poisson’s ratio of the tumor and host tissue does not affect qualitatively our results and the model can still provide a good fit to the experimental data. (TIF) [file pcbi.1012676.s008.tif]

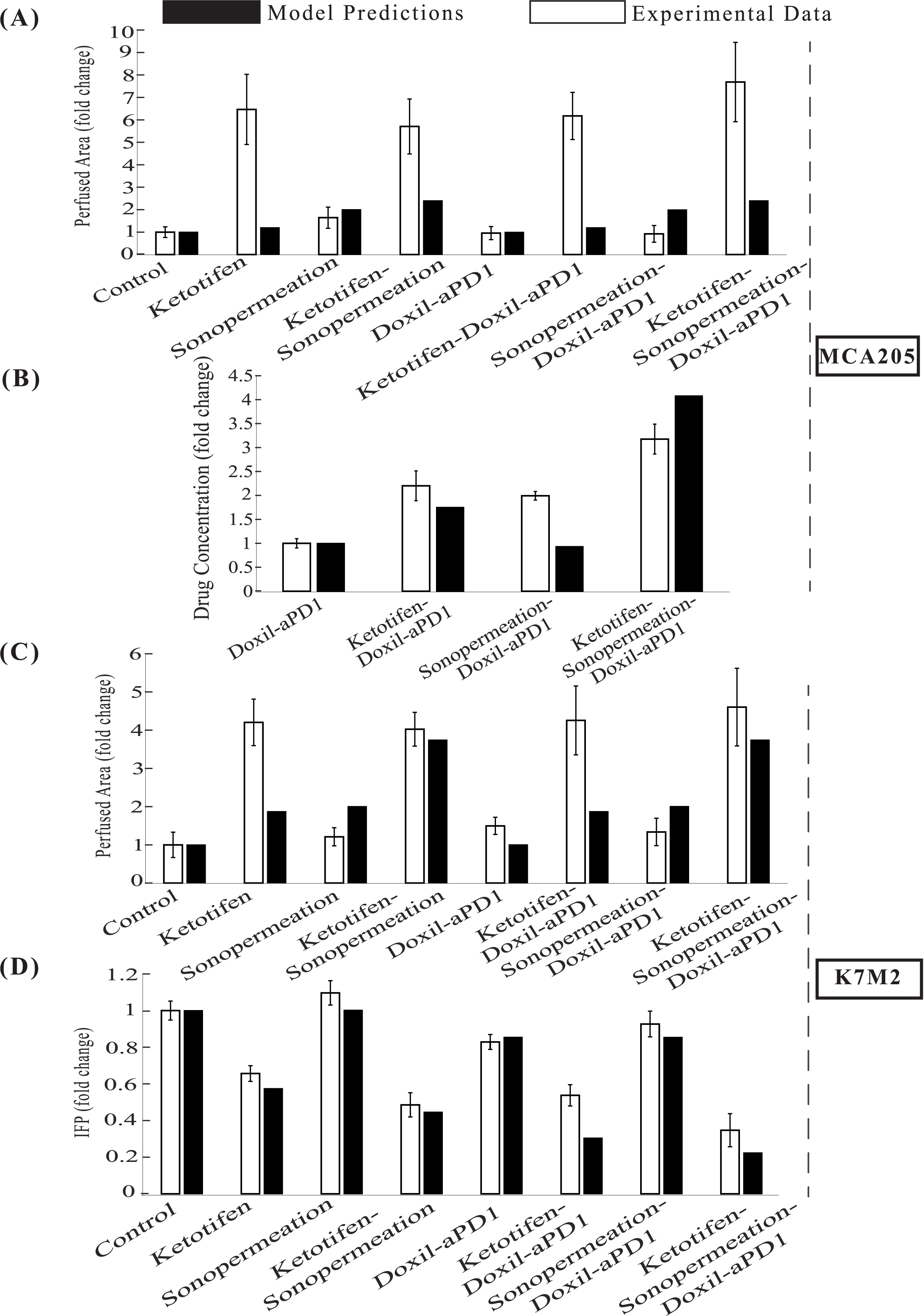

Supplement: S5 Fig — In these simulations, the host tissue is assigned a Poisson’s ratio of ν = 0.49, while the tumor tissue is modeled as incompressible, with a Poisson’s ratio of ν = 0.499999. The horizontal axis delineates the various treatment groups that were included in the experimental investigations: control, ketotifen, sonopermeation, ketotifen-sonopermeation, Doxil-aPD1, ketotifen-Doxil-aPD1, sonopermeation-Doxil-aPD1, ketotifen-sonopermeation-Doxil-aPD1. The vertical axis (y) for each instance varies between (A) Perfused Area and (B) Drug Concentration for MCA205 fibrosarcoma, (C) Perfused Area and (D) Interstitial Fluid Pressure (IFP) for K7M2 osteosarcoma. Again, we find that changing the values of the Poisson’s ratio for the tumor and host tissue can still provide a good fit to the experimental data. We note that increasing the values of the Poisson’s ratio of the tumor and host tissue does not affect qualitatively our results and the model can still provide a good fit to the experimental data. (TIF) [file pcbi.1012676.s009.tif]

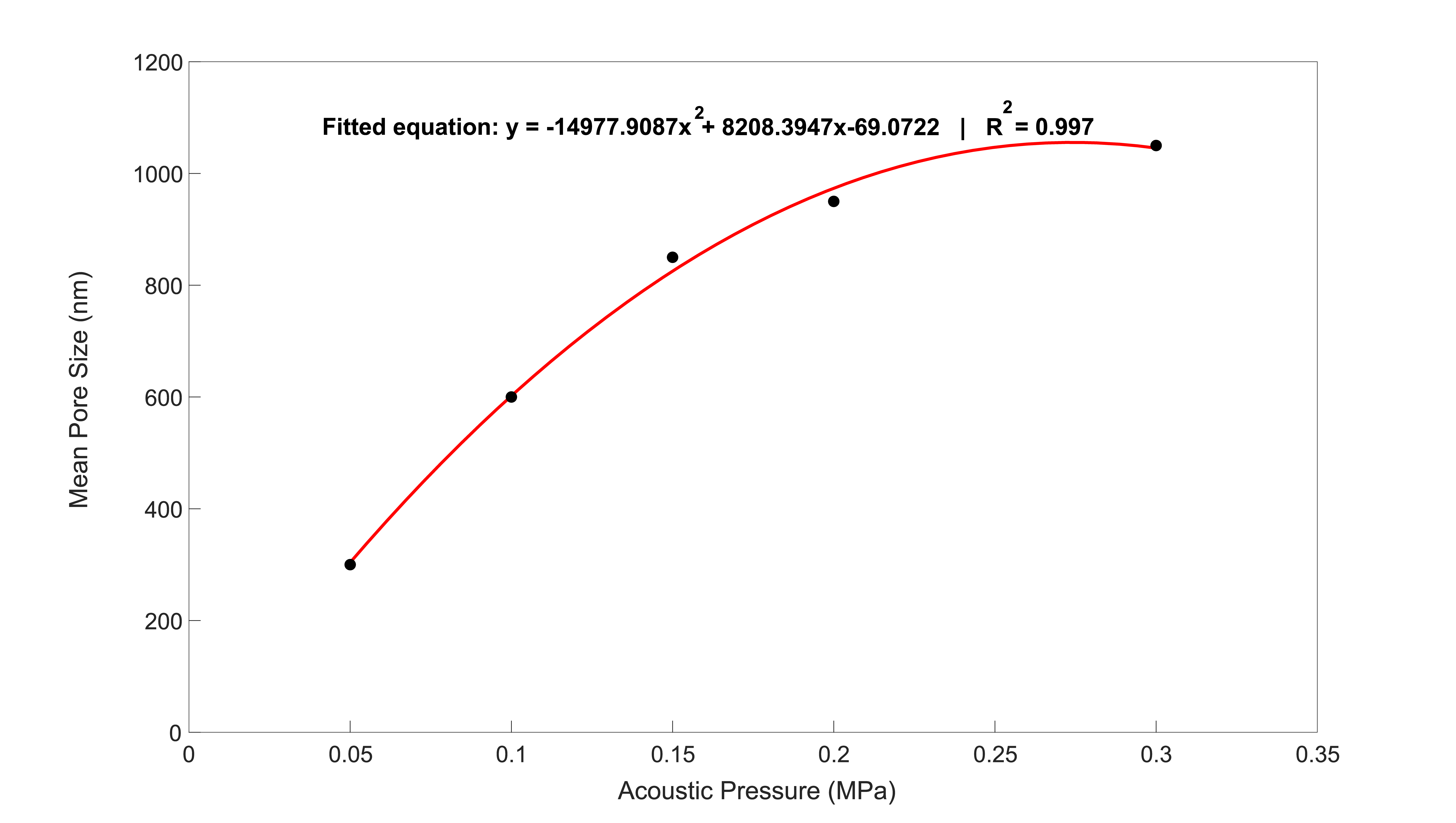

Supplement: S6 Fig — The second-degree polynomial curve was fitted to the data points, and the resulting equation is shown in the plot. (TIF) [file pcbi.1012676.s010.tif]
